# *In vivo* acoustic and photoacoustic focusing of circulating cells

**DOI:** 10.1038/srep21531

**Published:** 2016-03-16

**Authors:** Ekaterina I. Galanzha, Mark G. Viegas, Taras I. Malinsky, Alexander V. Melerzanov, Mazen A. Juratli, Mustafa Sarimollaoglu, Dmitry A. Nedosekin, Vladimir P. Zharov

**Affiliations:** 1 Arkansas Nanomedicine Center, University of Arkansas for Medical Sciences (UAMS), Little Rock, Arkansas 72205; 2 Bauman Moscow State Technical University, Moscow, Russia, 107005; 3 Moscow Institute of Physics and Technology (MIPT), Moscow Region, 141700, Russia

## Abstract

*In vivo* flow cytometry using vessels as natural tubes with native cell flows has revolutionized the study of rare circulating tumor cells in a complex blood background. However, the presence of many blood cells in the detection volume makes it difficult to count each cell in this volume. We introduce method for manipulation of circulating cells *in vivo* with the use of gradient acoustic forces induced by ultrasound and photoacoustic waves. In a murine model, we demonstrated cell trapping, redirecting and focusing in blood and lymph flow into a tight stream, noninvasive wall-free transportation of blood, and the potential for photoacoustic detection of sickle cells without labeling and of leukocytes targeted by functionalized nanoparticles. Integration of cell focusing with intravital imaging methods may provide a versatile biological tool for single-cell analysis in circulation, with a focus on *in vivo* needleless blood tests, and preclinical studies of human diseases in animal models.

Flow cytometry is a powerful biological tool for studying cell functional states, morphology, composition, proliferation, and protein expression that has led to many revolutionary discoveries in cell biology and medical diagnosis^1^,^2^,^3^,^4^,^5^,^6^. In conventional flow cytometry, cells flowing at a high rate (up to ~10^5^ cells/s) are accurately positioned into single file with a diameter of 5–10 μm. In combination with a tightly focused laser beam, this narrow sample stream creates a small interrogation volume that is analyzed by the collection of laser-induced fluorescent and scattered light with several photodetectors. This provides multiple parameters of fluorescence and scatter for each cell^1^. Nevertheless, invasive extraction of cells from a living organism may alter cell properties (e.g., signaling, epigenetic states, metabolic activities, morphology) and prevent the long-term study of cell properties and dynamics (e.g., cell–cell interactions, aggregation, rolling, or adhesion) in the natural biological environment^1^. *In vivo* flow cytometry using the blood and lymph vessels as natural tubes with native cell flow can overcome these problems^7^,^8^. This new-generation flow cytometry preferentially using photoacoustic (PA) and fluorescence detection methods has already demonstrated its unique utility for detecting extremely rare circulating tumor cells (CTCs), pathogens, and clots^7^,^8^,^9^,^10^,^11^,^12^,^13^,^14^,^15^,^16^,^17^. However, application of this powerful new tool for counting each normal and abnormal cell in the circulation is challenging because many (hundreds and more) red and white blood cells (RBCs and WBCs, respectively) can be simultaneously present in the laser-irradiated volume of relatively large (e.g., 50–300-μm diameter) blood vessels^8^,^18^. Small vessels and especially capillaries with single-file flexible RBCs are not quite suitable for *in vivo* flow cytometry because the majority of cells of interest, such as CTCs or WBCs with typical diameters of 12–25 μm and 8–12 μm, respectively, can be captured and thus cannot circulate in 5–7-μm-diameter capillaries, while the RBC rate is extremely low (e.g., 5–30 RBCs/s)^8^ for analytical application.

The problem of single cell counting *in vitro* was solved by cell manipulation and focusing using mechanical, optical, electrical, magnetic and other gradient forces^19^,^20^,^21^,^22^,^23^,^24^,^25^,^26^,^27^,^28^,^29^,^30^,^31^,^32^,^33^,^34^,^35^,^36^,^37^. However, adaptation of these methods to the *in vivo* condition, even in animal models^7^,^8^,^9^,^10^,^11^,^12^,^13^,^14^,^15^,^16^,^17^,^18^,^38^,^39^,^40^,^41^, faces many challenges due to the difficulty of accessing cells within deep vessels, limited control, the weakness of the forces used to overcome the drag forces acting on cells in bioflow (e.g., ~400 pN at a flow velocity of 5 mm/s)^38^, attenuation of gradient forces in biotissue, specific requirements on cells and medium, and possible harmful effects on cells. For example, optical tweezers are limited by the weakness of photonic forces (10–50 pN), the impossibility of strongly focusing the laser beam with an oil-immersion high-numerical-aperture 100× microobjective in deep tissues, and the possibility of damaging cells in the high-intensity light of the focal point. Hydrodynamic cell focusing using sheath fluids between two coaxial tubes *in vitro*^1^ requires invasive implantation of these tubes in vessels that could lead to circulation dysfunction. The use of vessel valves as natural nozzles was demonstrated in lymphatics only with specific anatomic valve structures^7^. The impact of high-intensity ultrasound on blood flow is invasive, ranging from vasodilation to vasoconstriction with the appearance of blood stasis and RBC aggregation^42^,^43^,^44^,^45^,^46^. None of these approaches have been explored for noninvasive controllable cell focusing in the blood circulation *in vivo*. As a result, despite the impressive application of *in vivo* flow cytometry for detecting single CTCs against the background of many blood cells in the detection volume^7^,^8^,^9^,^10^,^11^,^12^,^13^,^17^, the great potential of this method for counting individual blood cells and/or abnormal cells at high concentration has not yet been reported. However, it is important for many applications, including studies of the immune system, inflammatory processes, cell–cell interactions, cell rolling, aggregation, leukocytosis, and thrombotic and infectious disorders at the single-cell level^47^,^48^,^49^,^50^,^51^,^52^,^53^. Here we demonstrate methods for cell manipulation with an emphasis on focusing cells directly in blood and lymph vessels *in vivo* by means of gradient acoustic forces (Figs 1, 2, 3, 4, 5, 6, Supplementary Figs S1–15).

## Principle of acoustic cell focusing *in vivo* using ultrasound and photoacoustic (PA) waves

**Figure 1:**
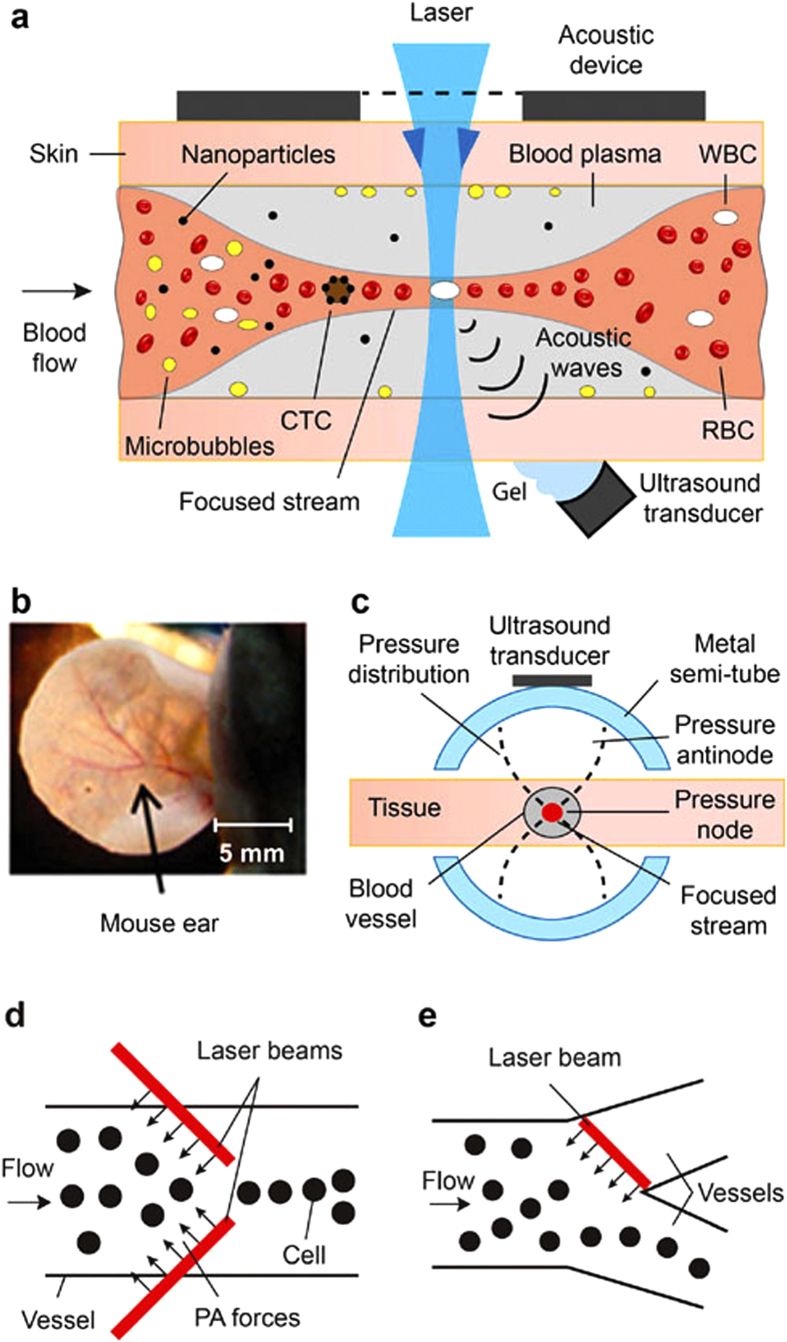
Principle of cell manipulation *in vivo*. (**a**) Schematic of *in vivo* flow cytometry with acoustic focusing and PA detection of circulating cells and nanoparticles. (**b**) Nude mouse ear-vessel model. (**c**) Cross-section of an acoustic resonator around a selected vessel in mouse ear skin. (**d**) Principle of PA focusing of flowing cells with two linear laser beams creating “virtual PA walls”. (**e**) Cell redirection between two blood vessels with a linear laser beam creating a virtual PA wall.

We hypothesize that acoustic cell focusing in nodal planes of standing ultrasound waves can be achieved *in vivo* directly in blood and lymph vessels (Fig. 1a). To overcome drag forces, strong acoustic forces can be created by using an external acoustic resonator that surrounds the vessel at an appropriate location, in particular, in an extremity or ear (Fig. 1b). Using this approach, we developed customized resonators consisting of two semitubes or one semitube and a flat substrate with attached piezoelectric transducers to excite standing wave with nodes in the center of the blood or lymph vessels (Fig. 1c). Ultrasound resonance frequencies in the acoustic resonators, which had different inner diameters—0.1 mm, 0.3 mm, and 1.5 mm—occurred at ~7.3 MHz, ~3 MHz, and 0.6 MHz, respectively. In selected experiments, we used a planar acoustic resonator with a commercial ultrasound source having a flat applicator and frequencies of 1 MHz and 3 MHz.

In the PA method, instead of piezoelectric transducers, acoustic waves are generated by laser through PA effects^8^ in the absorbing liquid around the cells, which push cells away or trap them in a laser beam with a ring configuration^31^,^32^,^33^,^34^. The PA forces can be significantly (≥10-fold) enhanced up to a level of 1–10 nN (i.e., three orders of magnitude higher than in optical tweezers) by the generation of nanobubbles in overheated absorbing zones^34^. We hypothesize that PA cell focusing can be achieved by creating virtual PA walls with one or two linear laser beams (Fig. 1d,e). A virtual PA wall in a vessel bifurcation zone can also redirect cells between the two vessels (Fig. 1e). Creation of the necessary PA effects is based on the use of a spectral range at which laser radiation is absorbed by natural blood plasma components (e.g., strong water absorption at 1.45 μm or 1.9–2.1 μm)^31^,^32^ or, when it is appropriate, on the administration of absorbing dyes approved for use in humans (e.g., indocyanine green [ICG])^54^.

## Verification of cell manipulation *in vitro*

**Figure 2:**
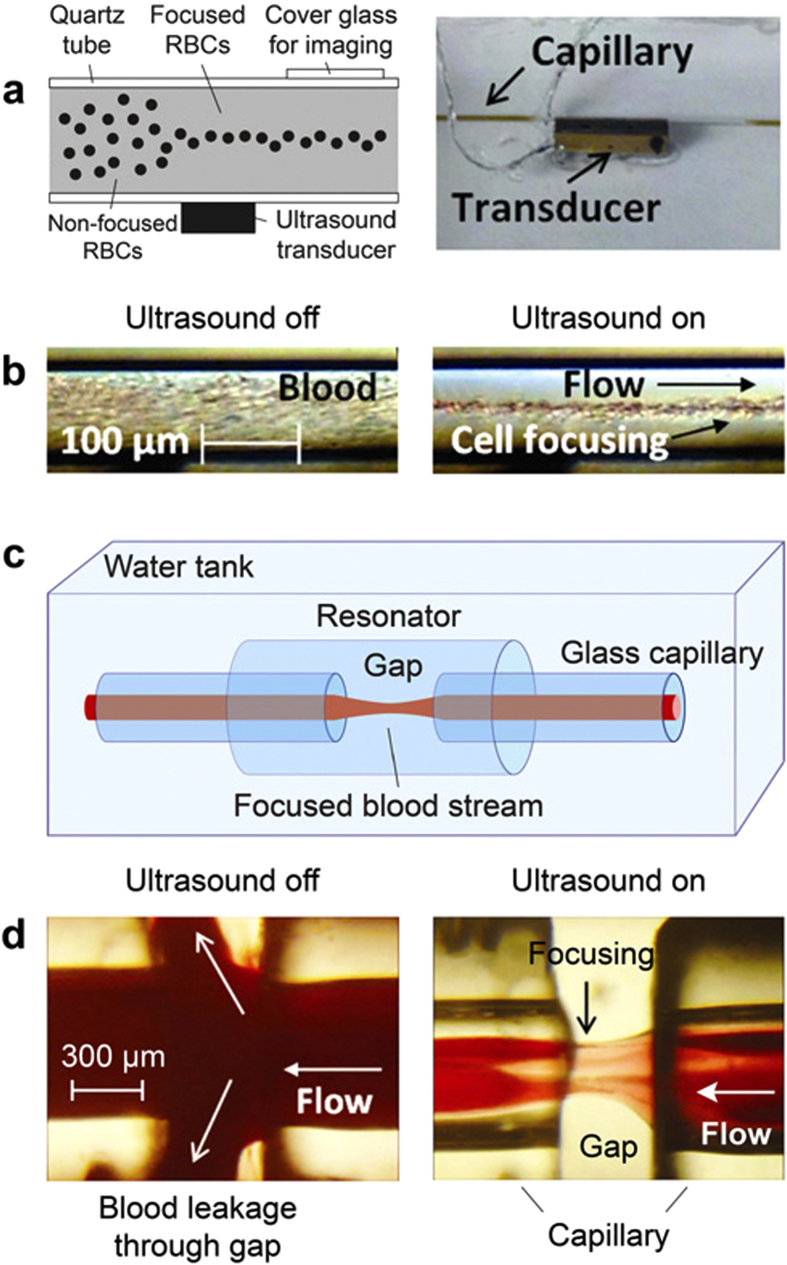
*In vitro* acoustic focusing of blood cells in flow. (**a**) Schematic of acoustic focusing (**left**) and experimental setup using a quartz capillary with a 100-μm inner diameter (**right**). (**b**) Distribution of mouse RBCs in flow before (**left**) and during (**right**) the application of ultrasound Ultrasound parameters: frequency, 7.29 MHz; intensity 0.5 W/cm^2^. (**c**) Schematic of the acoustic focusing of blood flow in the gap between two capillaries. (**d**) Leakage of blood flow between two capillaries (**left**) and acoustic canalization of blood flow in the gap between two capillaries (**right**). Ultrasound parameters: frequency, 0.6 MHz; intensity, 1.8 W/cm^2^.

**Figure 3:**
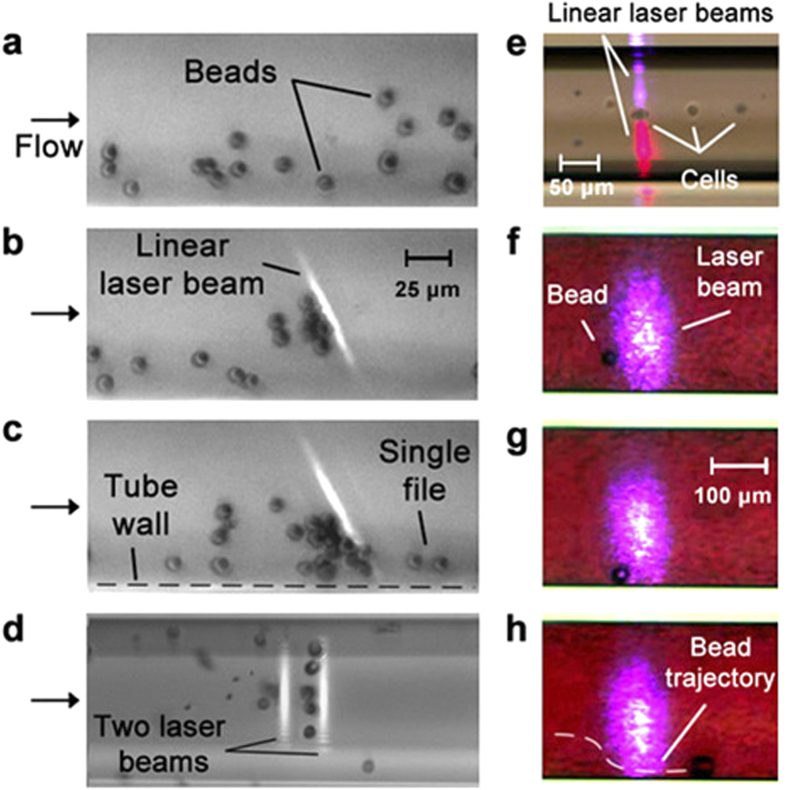
PA manipulation of beads and cancer cells *in vitro*. Polystyrene beads (**a–d,f–h)** and melanoma cells **(e)** were manipulated *in vitro* in a glass capillary filled with ICG in water (**a–d**: 6.8–μm beads) and in mouse blood (**f–h**): 25–μm beads with the use of linear laser beams of different spatial configurations. Laser parameters: wavelength, 820 nm (**a–c,f–h**); 671 nm (bottom) and 820 nm (top) (**e**); 671 nm (first) and 820 nm (second) (**d**); pulse width, 8 ns; pulse rate, 10 kHz; distance between two beams 20 μm (**d**); pulse energy, 5 μJ (energy fluence, 0.02–0.1 J/cm^2^).

The phenomenological models described above were verified first *in vitro* with the use of a flow module with glass capillaries of different shapes (Figs 1, 2, 3, Supplementary Figs S1–5). To create acoustically focused flow, we attached a 1 × 1 × 8-mm (width, thickness, length) piezoelectric transducer (PZT-4) to the external surface of a glass capillary to provide the ultrasound source inline contact with the glass capillary outer wall (Fig. 2a). The drive frequency was optimized by changing the frequency and voltage amplitude of the electrical generator; simultaneous control of the maximal amplitude of acoustic waves in the capillary was achieved with the ultrasound transducer (Methods). Using this technique, we demonstrated focusing of mouse RBCs over a considerable length of the flow chamber (Fig. 2b, Supplementary Movie S1) at flow velocities up to 10 mm/s. We also observed that acoustic focusing of blood flow makes it possible to transport blood across a gap between two capillaries (Fig. 2c, Supplementary Movie S2). Indeed, without the acoustic field, the suction effect created by the flow module was weak, allowing blood to escape through gaps between the capillaries into the external volume (Fig. 2d, left); in contrast, with the acoustic wave in place at the same flow module parameters, blood passed between the capillaries with no substantial loss (Fig. 2d, right).

For PA focusing, we used a glass capillary with a square cross-section located between the glass slide and the cover slip or a circular capillary embedded in glycerol to minimize optical distortion and light scattering (Supplementary Fig. S2). The capillary was filled either with a water solution containing ICG in a concentration of 5 μg/mL and 6.8-μm-diameter beads or mouse blood with ICG at similar concentration and 25-μm-diameter beads. The capillary was irradiated with one or two linear laser beams with adjustable spatial orientation (Supplementary Movie S3) and energy at wavelengths of 671 nm or 820 nm within the absorption spectrum of ICG^54^. We observed that a linear 4.5 × 80-μm laser beam at 820 nm with a slightly nonperpendicular orientation to the direction of flow blocked the central part of the capillary, thus preventing the 6.8-μm beads from moving through the beam at 4 ± 1 mm/s (Fig. 3a,b). As a result, beads moved along the beam, bent around it, and finally bypassed it in single file through a small slit (10 ± 3 μm) between the beam and the capillary wall (Fig. 3c, Supplementary Movie S4). This finding also suggests the feasibility of cell redirection (Fig. 1e). Two parallel beams across the capillary more effectively stopped beads moving at up to 7 mm/s. The first beam inhibited a bead’s movement, thus facilitating its capture by the second beam (Fig. 3d). The two beams with a small (12 ± 3.5 μm) gap between them propelled 12–16-μm-diameter melanoma cells forward in single file only through this gap (Fig. 3e). The multiple parallel laser beams along the capillary can divide the flow into separate subflows between individual laser beams. Beads of cells can bend around the laser beam. Indeed, the effect of bending the trajectory of the beads around the linear laser beam was also observed in mouse blood flow (Fig. 3f–h). We experimentally confirmed our theoretical model^33^,^34^ predicting two types of PA forces: near-field forces, pushing particles away from the laser beam due to strong local acoustic pressures; and far-field forces, pulling particles toward the laser beam due to acoustic and thermodynamic suction effects^31^,^32^,^33^,^34^. In the dynamic mode, these forces can lead to particle oscillation (Supplementary Movie S5). Laser-induced strong PA waves near the laser beam can travel along the glass capillary as an acoustic waveguide and push the bubbles away against flow (Supplementary Movie S6). It should be noted that the acoustic waveguide effect could not happen *in vivo*, or at least would be weak, because of acoustic impedance mismatch for the vessel wall compared to the glass wall *in vitro*.

## Cell focusing *in vivo*

**Figure 4:**
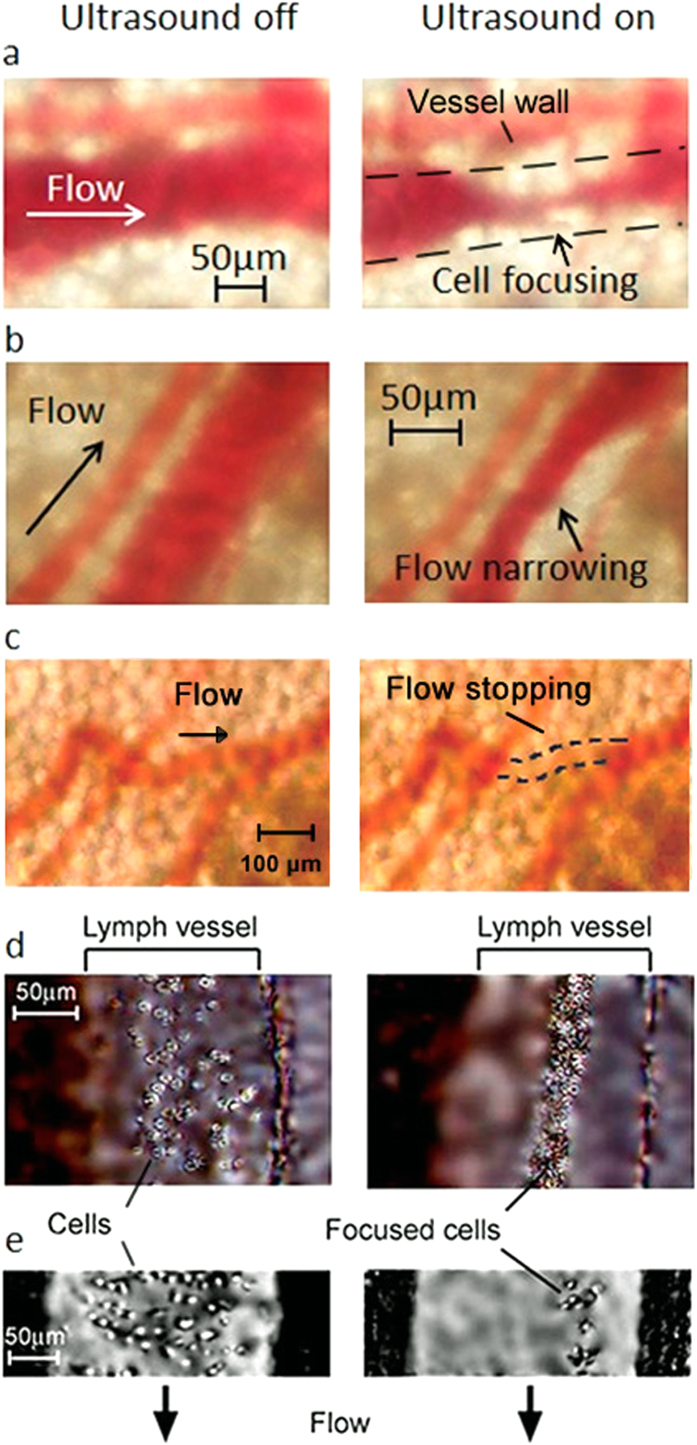
*In vivo* cell focusing in blood and lymph flow in living animals using acoustic waves. (**a**) Blood flow before (**left**) and after (**right**) application of ultrasound in a mouse ear vessel. The dashed lines mark the vessel’s boundary. (**b**) Asymmetric displacement of blood flow to a vessel wall under ultrasound action (**right**), compared to control (**left**). (**c)** Acoustic stopping of blood flow in a localized zone (**right**), compared to control (**left**). (**d,e**) Acoustic focusing of cells (WBCs) in an 180-μm-diameter mouse mesenteric lymph vessel under the influence of acoustic standing waves in two different vessels. Ultrasound parameters: frequency, ~3 MHz; intensity, 0.4 W/cm^2^ (**d**) and 1 W/cm^2^ (**e**). Average flow velocities: 5 mm/s (blood); 1.5 mm/s (lymph).

**Figure 5:**
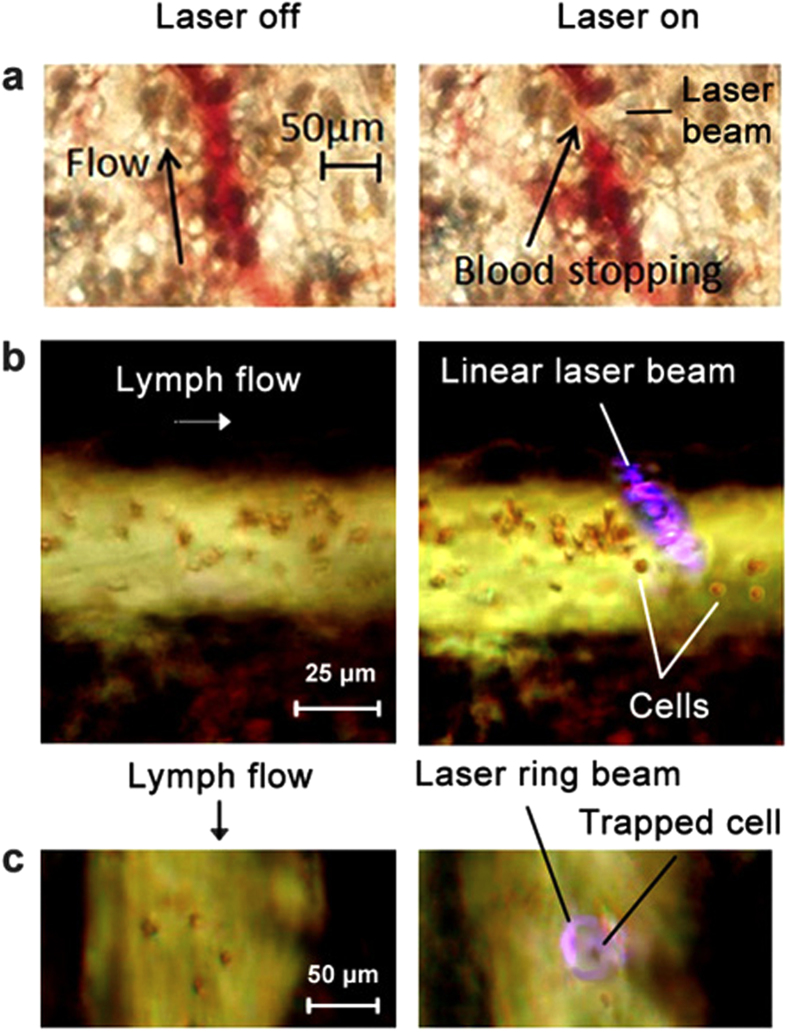
PA manipulation of cells *in vivo*. (**a**) Blood flow before (**left**) and after (**right**) laser irradiation in the area of mouse ear vessels indicated by the arrow. (**b**) Cells in lymph flow of a mouse mesentery vessel before (**left**) and after (**right**) irradiation with a linear laser beam. The passage of the laser beam through vessels blurred it into an ellipsoidal shape as seen in image due to light scattering in the tissue. (**c**) Trapping of a single cell in a mouse mesenteric lymph vessel with a ring-shaped laser beam **(right**). Laser parameters: wavelength, 532 nm (**a**) and 820 nm (**b,c**); energy fluence, 0.7 J/cm^2^ (**a**) and 60 mJ/cm^2^ (**b,c**).

We explored cell focusing in blood and lymph flow in the ear and mesentery of mice, using gradient acoustic forces induced by ultrasound and PA waves (Figs 4 and 5, Supplementary Figs 4–6). Using the schematic in Fig. 1c, we acoustically manipulated blood flow in a 60 ± 10 μm-diameter mouse-ear blood vessel, including narrowing of blood flow in the vessel’s center without changing the vessel’s diameter (Fig. 4a, Supplementary Movie S7 and Figs S7 and S12), asymmetric displacement of flow to one vessel wall (Fig. 4b), and almost complete cessation of blood flow (Fig. 4c, Supplementary Movie S8). Specifically, the focused area with closely packed RBCs was separated from cell-free plasma situated between it and the vessel walls (Fig. 4a, right, dashed lines) as RBCs were pushed out from pressure antinodes. In a ~50-μm-diameter vein, where flow velocity is typically 4 ± 1 mm/s^7^,^16^,^17^,^18^, spatially changing the trajectory of blood flow was initiated at an ultrasound intensity of 0.2 ± 0.07 W/cm^2^, with the appearance of more profound effects at an intensity of 0.6 ± 0.1 W/cm^2^ (a peak negative pressure≤0.1–0.3 MPa) and an ultrasound frequency of 3 ± 0.1 MHz. The typical time for the appearance of these phenomena was 1–2 s after the ultrasound was switched on. The transition from focusing cells in the vessel’s center to concentrating them asymmetrically near the vessel wall (Fig. 4b, right) was achieved by a tiny adjustment of the acoustic resonator’s spatial position. Blood flow was completely stopped by changing the spatial position of the ultrasound source when the antinodes were likely oriented perpendicular to the vessel’s axis (Fig. 4c, right). Eventually, in a 70 ± 5-μm-diameter ear vessel, we achieved the narrowing of blood flow to a 10 ± 3-μm-wide stream (i.e., 7-fold), which is comparable to the size of single cells. Similar results were obtained in 150 ± 25-μm-diameter lymph vessels of mouse mesentery (Fig. 4d,e, Supplementary Fig. S11). The observed focusing phenomena in both blood and lymph vessels were relatively stable at least for 10–15 minutes and reversible during multiple (up to 10) on-off switching cycles of the ultrasound. With a longer period, the spatial positions of the focused stream in the vessel can be slightly changed due to likely physiological motion requiring a tiny further spatial adjustment of the acoustic resonator. Although we demonstrated acoustic focusing on the vessel’s length up to 1 mm, in many cases, focusing phenomena were more localized. These can be explained by the vessel’s not quite parallel position and the axial position of the acoustic resonator due to the vessel’s heterogeneous anatomic structure and spatial location compared to the ideal cylindrical configuration of an artificial tube and resonators *in vitro*^28^,^29^.

Cell manipulation *in vivo* using a PA source of acoustic waves was validated with the schematic shown in Fig. 1d. The manipulation included reversible blood flow cessation as 30–40-μm-diameter blood microvessels in mouse ear were irradiated with a 532-nm-wavelength laser with a linear beam geometry (4.5 × 80 μm) without any contrast agents (Fig. 5a, Supplementary Movie S9, Fig. S13). This phenomenon is the result of creating a strong asymmetric gradient acoustic force during the laser irradiation of RBCs, which have strong absorption at 532 nm. It is in line with our previous finding *in vitro* when spatially asymmetric laser irradiation of individual particles and cells pushed them in the opposite direction as a result of localized reactive acoustic effects^31^,^32^,^33^,^34^. It should be noted that, in Movie S9, the linear laser beam likely partly overlapped this small vessel on the left side during exposure of the main 40–45-μm diameter vessel, leading to blood flow arrest also in this small vessel, which ceased when the laser was off. In blood flow, the linear beam shape provided similar collective effects for many RBCs, whereby cells were pushed away from the laser beam when the cell during its movement touched the boundary of the laser beam. However, to achieve these effects in blood, we needed to use relatively high laser energy fluences, up to 0.3 J/cm^2^, that sometimes led to RBC damage, especially during spontaneous formation of RBC aggregates with stronger localized absorption. Indeed, according to the laser safety standard^55^, for a 532-nm wavelength, a safe fluence is 20 mJ/cm^2^, while in the near-infrared (NIR) window of tissue transparency (650–1,100 nm), where RBC absorption is significantly lower, a safe fluence is 100 mJ/cm^2^ with a 1,064-nm laser and ~75 mJ/cm^2^ with an 820-nm laser. That is why in our next studies we used NIR lasers at 671 nm, 820 nm, and 1064 nm. Because of 10–20-fold lower RBC absorption at these wavelengths, we injected in a tail vein 10 μL of ICG solution at a concentration of 5 mg/mL, which is equal to 2 mg/kg of mouse body weight (i.e., within the FDA-recommended ICG dosing in humans)^54^. Approximately 20 minutes later, ICG appeared in lymph flow and then in tissue around vessels (Supplementary Fig. S14). This led to increase the PA effects in in surrounding blood plasma compared to the cells themselves. In turn, it led to generating strong gradient acoustic forces in plasma that prevented cell movement across the beam. In particular, there were no cells behind the linear laser beam, with many cells in front of the laser beam (Fig. 5b, right). As a result, cells moved along the beam, bent around it, and finally bypassed it in single file through the gap (20 ± 5 μm) between the beam and the lymph vessel wall. Using a ring-shaped laser beam with a 30 ± 3-μm internal diameter enabled stably capturing a single cell inside the light ring (Fig. 5c, right), as we demonstrated previously *in vitro*^34^. The intravenous injection of 50 μL of a gold nanoshell solution (concentration, 5 × 10^12^ nanoparticles/mL) with stronger near-infrared absorption than ICG led to more profound PA effects in particular narrowing of blood flow in the gap between two laser beams (Fig. 1d at 671 nm and 820 nm, Supplementary Movie S10).

## *In vivo* PA flow cytometry (PAFC) with acoustic focusing of cells in blood flow

We applied acoustic cell focusing schematics (Figs 1a and 4b) to *in vivo* PAFC, which is based on the irradiation of selected vessels by laser pulses followed by detection of laser-induced acoustic waves from absorbing targets with ultrasound transducer^8^,^17^. Possible interference between the ultrasound sources used for acoustic focusing with the PAFC detection system was decreased by using different laser pulse rates (1–10 kHz), ultrasound frequencies (0.6–3 MHz) and spectral PA signal filtration. Without acoustic focusing, the laser generated background PA signals determined by the number of RBCs in the irradiated volume. This number depends on vessel size and the spatial resolution of the PAFC instrument, which can vary from hundreds to thousands of cells^8^. Conventional PAFC can count rare strongly pigmented or labeled cells only (e.g., melanoma with melanin^8^, infected RBCs with hemozoin produced by malaria parasites^53^ or CTCs with nanoparticles^8^) when only one such cell is present in the detection volume and the signal from this cell exceeds the background signals generated by the many RBCs in the same volume.

However, PAFC can miss low-absorbing cells or cannot count many cells in the detection volume because of overlapping of signals. This is typical for sickle disease, in which the abundance of rigid sickle cells with abnormal properties causes blood flow dysfunction and eventually sickle crises^8^,^41^. Sickle cells’ differences from normal RBCs in shape, in larger size, and in lower local hemoglobin concentration, as we discovered, cause the cells to generate ~2-fold lower PA signal amplitudes^8^. This was demonstrated *in vitro* in a capillary tube by comparison of PA signals from normal RBCs and sickle cells collected from control mice and genetically modified mouse models expressing human sickle hemoglobin^41^, respectively (Supplementary Fig. S16).

**Figure 6:**
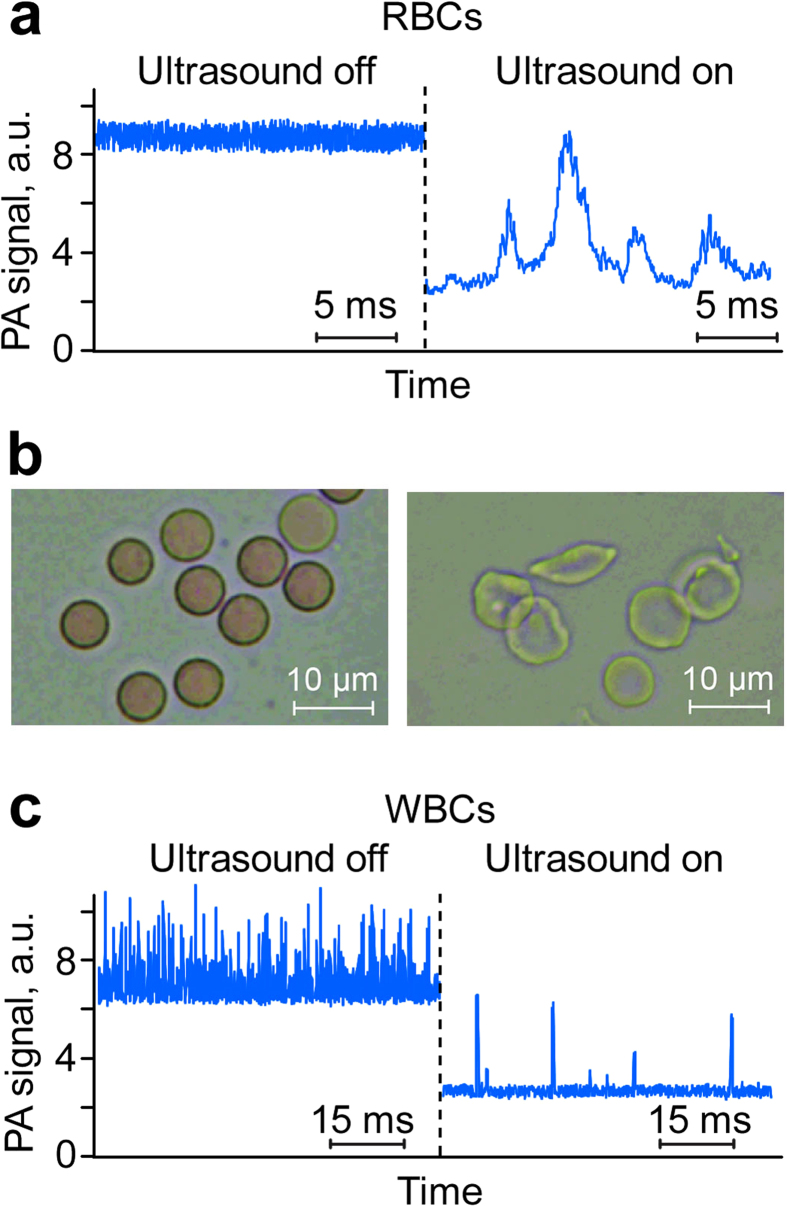
*In vivo* PAFC with acoustic cell focusing. (**a**) *In vivo* label-free PA detection of individual RBCs in focused flow **(right)** compared to nonfocused flow (**left**) using a planar acoustic resonator. **(b)** Optical (transmission) images of normal **(left**) and sickle **(right)** RBCs. **(c)** *In vivo* time-resolved PA detection of individual WBCs (leukocytes) molecularly targeted by gold nanorods (GNRs) conjugated with antibody to CD45 receptors in focused blood flow in mouse ear microvessels **(right)** compared to the overlapping peaks from WBCs in nonfocused flow **(left).** Laser parameters: wavelength/fluence, 532 nm/40 mJ/cm^2^ (**a**) and 820 nm/50 mJ/cm^2^ (**c**). Ultrasound parameters: frequency, 3 MHz; intensity, 0.5 W/cm^2^.

To distinguish PA peaks from individual RBCs *in vivo*, we applied acoustic focusing to 60 ± 5-μm-diameter ear blood vessels of the genetically modified mouse model expressing human sickle hemoglobin^41^. As a result, in label-free mode at a laser wavelength of 532 nm, instead of a constant PA background as in conventional PAFC mode^8^ (Fig. 6a, left), we observed clearly distinguishable PA peaks from individual RBCs, with estimated distances between them of 4–10 μm in well-focused flow (Fig. 6a, right). In contrast to the relatively homogeneous PA signal amplitude distribution of normal RBCs with an amplitude variation within 10–30%^8^, a more heterogeneous PA signal amplitude distribution was observed in sickle cell mouse model, with the presence large peaks against the background of many peaks with lower amplitudes associated with normal RBCs and sickle cells, respectively (Supplementary Fig. S16). Indeed, *in vitro* analysis of collected blood samples revealed the presence of RBCs with specific sickle-associated RBC shapes (Fig. 6b). These findings point to the potential of *in vivo* PAFC with cell focusing after further optimization to study human sickle cell disease in animal models.

Cell focusing has also been used for PA detection of individual WBCs. Because of the weak absorption by WBCs compared to that of RBCs, we molecularly targeted WBCs directly in the bloodstream by using strongly absorbing gold nanorods (GNRs) as high-contrast PA agents^8^ to enhance PAFC’s sensitivity. GNRs measuring 10 × 55 nm and having maximum absorption at 820 nm were conjugated with antibodies against the WBC receptor CD45. Intravenous injection of the GNR–antibody conjugates led to labeling of WBCs (~0.4% among RBCs) directly in the bloodstream over 30 min. At a laser wavelength of 820 nm in an 60 ± 8-μm-diameter vessel, many closely located and partly overlapping PA signal peaks were observed because of the random appearance simultaneously of several WBCs (at least two or three) in the detection volume (Fig. 6c, left). However, after acoustic narrowing (focusing) of the flow, the WBC peaks were completely separated because not more than one WBC was present in the detection volume each time. Under the conditions of our experiments, PA signals from labeled WBCs were much higher than those from RBCs. This finding was verified by comparison of PA signals from normal RBCs, WBCs, GNRs, WBCs with GNRs, and blood with targeted WBCs (Supplementary Fig. S17). This suggests that background signal from RBCs, which is a serious problem in conventional *in vivo* flow cytometry^8^, was successfully reduced. As work on *in vivo* cell labeling directly in the bloodstream for markers of interest progresses, such focusing techniques should play an important part in providing sensitive and accurate measurements of the labeling efficiency of cells with nanoparticles.

## Study of cell viability

In most experiments, we used an ultrasound intensity within the safety standard for peripheral vessels (720 mW/cm^2^)^46^, which was sufficient to produce cell-focusing effects. Nevertheless, because of the possibility of localized resonance concentrating of ultrasound energy, we tested mouse WBC viability at different ultrasound intensities (0.7 W/cm^2^ and 2.2 W/cm^2^) and exposure times (10 s, 5 min, and 30 min), using standard cell viability assays (trypan blue, Annexin V-PI, and CellTiter-Glo kits) under similar conditions used for cell focusing (Methods, Supplementary Information, Fig. S15a–c). Specifically, WBCs were extracted from mouse blood according to a well-established protocol and then exposed to ultrasound in an Eppendorf container and a glass capillary with a 300-μm inner diameter with a commercial ultrasound source (Sonicator 730) and customized acoustic resonator (Fig. 1c, Fig. S12). WBCs were found to be 92 ± 2.7% viable after 30 min at room temperature. We did not observe significant changes in viability (Supplementary Fig. S15d,e) with the trypan blue and Annexin V-PI assays, while the CellTiter-Glo kit revealed slight (~14%) inhibition of cell metabolic activity (decreased ATP). The temperature rise in the sample was found to be <1 °C. Thus, any possible bioeffects in this study were considered to be nonthermal. This is in line with FDA data indicating an excellent safety record for ultrasound imaging under similar condition over the many years it has been used^46^. In addition, according to the maker of the Attune cytometer (Life Technologies/Thermo Fisher Scientific), which uses acoustic focusing *in vitro*, acoustic forces are safe for live cells, and acoustic focusing does not significantly affect cell viability^56^.

## Discussion

Both ultrasound and PA techniques have previously been explored only *in vitro* for the manipulation of cells with acoustic and PA tweezers^27^,^31^,^32^,^33^,^34^. Standing waves also used in an acoustic resonator keep the cells stationary in the same position or concentrate them in the center of the stream in a flow cytometer^28^,^29^,^30^. Here we have introduced noninvasive methods using gradient acoustic forces induced by ultrasound and PA waves for controllable manipulation and especially focusing of cell flow 5–10-fold to a tight 5–10-μm-diameter stream in 50–70-μm-diameter blood vessels and in 150–200-μm-diameter lymph vessels with flow velocities of 5–7 mm/s and 1–3 mm/s, respectively^8^. We demonstrated cell trapping, redirecting and focusing in biological flows with the potential to accomplish cell counting and wall-free transportation. A focus of our study was the development of a new, versatile biological tool for cell manipulation directly in circulation in natural biological environments using an *in vivo* flow cytometry platform with various detection methods (e.g., PA, photothermal, fluorescent, or Raman)^7^ and animal models of human diseases including sickle cell anemia^41^,^47^, beta-thalassemia^39^, infections^7^,^53^, diabetes, and immune system disorders. In particular, this research tool can provide insights into immune system dysfunction through real-time enumeration of individual WBC subpopulations (e.g., neutrophils, lymphocytes, monocytes), detection of abnormal RBC subsets (e.g., sickle cells, aggregates, or infected by malaria parasites), study of blood cell epigenetics, rheology and chemistry (*in vivo* needleless blood test), or analysis of an unknown cell composition in lymph flow. Indeed, because of the difficulties of lymph sampling, basic knowledge of biological processes in lymphatics is lacking, including quantities of lymphatic macromolecules (e.g., cytokines, fatty acids) and cells (e.g., macrophages, B and T cells) and their interactions with each other and with endothelial cells^49^,^50^,^51^,^52^. In addition, identification of WBC aggregates by means of closely located PA peaks^8^ could be important for studying neutrophil–platelet interactions involved in several thrombotic and inflammatory disorders and in leukocytosis^18^. The focusing of flow near a vessel’s wall or center can, respectively, enhance or prevent the interaction of flowing cells of interest with endothelial cells, thus providing a controlled microenvironment for basic studies of rolling effects, adhesion, and cell–cell interactions that are important for understanding the progression of cancer metastasis, infections, and cardiovascular diseases.

Accurate cell positioning in single file in the center of a flowing stream enables reducing the number of blood cells in the detection volume. In turn, it minimizes background signals that are a problem in conventional *in vivo* flow cytometry with many blood cells in the detection volume producing overlapping signals. Besides, the center of flow moves at the highest velocity, and variation in velocity near the center is low, compared to the variation in more slowly moving blood plasma near the wall. As a result, the presence each time of one or just few cells in a focused laser beam can more sensitively and accurately enumerate circulating individual normal and abnormal cells (e.g., malignant, infected), extracellular vesicles, viruses and bacteria.

In the current studies, we confirmed high levels of acoustic and PA forces that allowed overcoming viscous drag forces (~4 × 10^–10^ N for RBC flow at velocity of 3–5 mm/s)^38^. In particular, the schematic in Fig. 3d allowed the trapping of cells and beads at the maximum available velocity (up to 30–50 cm/s) that suggested PA forces ≥6 nN, which is line with our previous estimate that PA forces can reach 10–20 nN^33^,^34^. Acoustic waves have also been shown to create strong gradient forces of ~10 nN^27^, i.e., three orders of magnitude greater than optical tweezers^19^. Thus, both acoustic and PA forces can easily exceed the drag forces of flow at a blood velocity of 5–10 mm/s in 50–70-μm-diameter blood vessels^8^.

PA forces can be enhanced by use of ICG in a concentration ≤2 mg/kg (which is approved for use in animals and humans)^54^, together with a low laser energy fluence level within the safety standard (35–100 mJ/cm^2^ in the NIR range of 650–1,100 nm)^55^. These unprecedented results cannot be achieved with existing methods, which permit only blood cell stopping/banding with the use of ultrasound^43^ or trapping of slowly moving cells in a small blood capillary with optical tweezers^38^.

In the case of a living animal model, the acoustic resonator (Fig. 1c) could be applied to the ear, extremities, or tail. PA manipulation requires a simpler schematic than is the case with an ultrasound transducer, with greater flexibility in the spatial localization of the gradient forces generated by remote laser sources (e.g., with a linear or ring beam geometry). Besides focusing a laser beam near cells, the forces produced by irradiation of the boundary of absorbing beads or cells (e.g., RBCs) can create asymmetrical forces pushing the cells away from the laser beam.

Nevertheless, more profound focusing effects have been achieved with ultrasound, although they require acoustic contact of the transducer with a vessel (e.g., through water or gel) and an external acoustic resonator. We demonstrated acoustic cell focusing in 50–70-μm-diameter vessels at depths of up to 150–200 μm, which is difficult to achieve with other methods, particularly optical tweezers operating at a much lower depth^38^. Both ultrasound and PA techniques can potentially assess larger, deeper vessels, in particular, the ~1-mm-diameter aorta in a mouse model^8^, which lies a few millimeters deep and has a flow rate up to 0.1–1 mL/s. Thus, PAFC can potentially enumerate individual cells at rates up to 10^8^ cells/s, which is 3–4 orders of magnitude higher than in conventional flow cytometry^1^. We believe that acoustically focusing cells into a narrow stream could be possible in 0.5–1-mm-diameter vessels by enhancing acoustic power. The effects of focusing and the impact of this energy on normal biotissues, however, require further study.

Surprisingly, we determined that the applicator from the commercial ultrasound device (Sonicator 730) provided focusing effects even without the acoustic resonator at some applicator locations above vessels. This could be explained by the interference of incident acoustic waves or waves reflected off surrounding structures (e.g., bones) or the mechanical holder and a flat glass substrate (planar resonator). However, the observed effects were less profound and more unstable, and required relatively high-intensity ultrasound (up to 2–3 W/cm^2^).

Acoustic forces can move an object toward either a node or an antinode of a standing wave, depending on the object’s size, density and compressibility^27^,^28^,^29^,^30^. Specifically, these forces are proportional to the third power of an object’s size^27^. The Stokes drag force, which resists the acoustic force, is linearly proportional to size, so that small objects will be focused less effectively or move more slowly than large objects. Thus, relatively large RBCs, WBCs, or CTCs will gather in pressure nodes (e.g., in the center of a blood or lymph vessel). In contrast, small, low-density objects (e.g., platelets, microbubbles, hollow liposomes, lipid particles) will gather in pressure antinodes (i.e., close to a vessel’s side walls)^28^. Thus, acoustic forces could separate cells concentrating in nodes from unbound contrast agents (e.g., dyes or nanoparticles). Although this hypothesis is supported by data from other groups indicating the possibility of acoustically separating WBCs, RBCs, malaria parasites and particles^57^, this hypothesis requires further verification.

In all conditions relevant to cell focusing, we observed no significant changes in cell viability at ultrasound parameters used during 5 min and even 30 min of exposure. It should be noted that in actual measurements, circulating cells in the peripheral blood vessels are exposed for a much shorter time (a few seconds). Our safety-related data are in line with FDA data supporting the excellent safety record of ultrasound imaging using well-established safety standard for peripheral vessels (720 mW/cm^2^ at exposure time up to 500s) over many years^46^. In addition, the Attune cytometer routinely uses ultrasound waves at similar parameters for cell focusing *in vitro* without significant effects on cell viability.

Acoustic forces in our study were strong enough to stop blood flow and to canalize it into narrower streams. High-intensity focused ultrasound (2500–3000 W/cm^2^, 2–3.5 MHz) has been shown to be effective in inducing hemostasis in actively bleeding, injured solid organs and blood vessels through ultrasound-induced invasive thermal (temperature elevation up to 70–80 °C) and mechanical (cavitation and streaming) phenomena leading to cell membrane damage, protein denaturation or tissue coagulation. In our approach, cessation of blood flow and flow canalization in the absence of a vessel wall require a much lower ultrasound intensity (≤0.7 W/cm^2^, i.e., three orders of magnitude lower) without thermal and mechanical effects^45^. There is a distinct gap between cell damage thresholds and the noninvasive intensities used in our study, which are typical for medical ultrasound imaging and long shown to be safe^46^. The ability to temporarily stop blood flow at a low intensity could be important for studying blood flow hemodynamics, rheology, blood-cell interactions with and without vessel walls, as well as the role of the blood circulation in tissue oxygenation or microinfarction. It could be also useful for understanding the interaction of low-intensity ultrasound (used in many diagnostic and therapeutic applications) with living tissues to determine safety limits and predict possible harmful or undesired impacts on blood and lymph flow and vessels.

## Methods

### Integrated photoacoustic (PA) flow cytometry (PAFC)

The schematic of PAFC has been described elsewhere^8^. Briefly, the principle of *in vivo* PAFC is based on irradiation of selected vessels with short laser pulses followed by time-resolved detection of laser-induced acoustic waves (referred to as PA signals) with an ultrasound transducer attached to the skin (Fig. 1a; Supplementary Figs 1 and 2). The PAFC setup was built on the platform of an Olympus IX81 inverted microscope and was equipped with high-pulse-repetition-rate nanosecond lasers with the following parameters: 1) wavelength, 532 nm; maximal pulse energy, 30 μJ; pulse width, 5 ns; and pulse-repetition rate, up to 100 kHz (model: LUCE 532, Bright Solutions, Cura Carpignano, Italy); 2) 671 nm, 35 μJ, 25 ns, and 100 kHz (model: QL671-500, CrystaLaser, Reno, NV); and 3) 820 nm, 75 μJ, 8 ns, and 30 kHz (model: LUCE 820, Bright Solutions). The selected PA contrast agents (below) exhibited peaks in absorption spectra coinciding with the laser wavelengths used (i.e., near 532 nm, 671 nm, and 820 nm, respectively). In each experiment, laser energy levels were adjusted, depending on the task. In most applications, the pulse-repetition rate for these lasers was 10 kHz. Laser pulses were triggered by a digital delay/pulse generator (DG645, Stanford Research Systems, Sunnyvale, CA).

The laser beams were navigated on selected blood vessels with an XY motorized stage with the use of a controller (model: STG4400ML, Conix Research, Inc., Springfield, OR) under optical imaging guidance. Laser radiation was focused into a linear configuration on the sample measuring from 4.5 μm × 40 μm to 8 μm × 80 μm by various customized optical schemes. In particular, we used the assembly of either aspheric (C560TME-C) or cylindrical (LJ1310-L1-C) lenses (Thorlabs Inc.) with optical focal lengths of 6–10 mm at working distances of 4 mm and 6 mm and a customized condenser with cylindrical lenses and a 40×, 0.65-NA microobjective with a focal length of 15 mm.

The PA signals, which have a bipolar shape and a typical duration of 0.5 μs, were detected by ultrasound transducers (an unfocused XMS-310 transducer with a 10-MHz frequency band or a focused V316-SM transducer with a 20-MHz frequency band; both from Panametrics-NDT/Olympus, Waltham, MA) and then amplified (model 5662 or 5678: Panametrics-NDT). The PA signals are digitized with a high-speed, analog-to-digital converter board, and then peak-to-peak amplitudes of the acquired PA signals are presented as PA signal traces (Fig. 6) in analogy to conventional flow cytometry^1^. Real-time and post-processing operations were performed with MATLAB v. 7.0.1 software (MathWorks, Natick, MA).

Signal-to-noise ratio (SNR) in PAFC is determined by the ratio of flash (transient) PA signals from single strongly absorbing objects (e.g., cell or nanoparticles) to the superposed background PA signals from RBCs in the detection volume and to noise of different origins (e.g., electronic, acoustic, fluctuating RBC numbers, instability of laser energy). To enhance PA signals from nonpigmented cells (e.g., WBCs), the cells are molecularly targeted by strongly absorbing nanoparticles.

In optical-resolution PAFC, resolution is determined by optical parameters, in particular, the minimal width of a focused linear laser beam. Due to strong light scattering in tissue, high spatial resolution at the level of 3–10 μm can be achieved in superficial 30–50-μm-diameter vessels. In acoustic-resolution PAFC, resolution in deeper tissue with strong light scattering depends on the ultrasound focal parameters (e.g., 20–100 μm at a frequency of 10–50 MHz, respectively).

To image blood vessels and to verify cell-focusing effects, the PAFC setup was equipped with a high-resolution optical (transmission) module. This module used a cooled, color CCD camera (model: DP72, Olympus-NDT), a high-speed (up to 40,000 frames per second) CMOS digital camera (model MV-D1024-160-CL8: Photonfocus AG, Lachen, Switzerland), and a Cascade 650 CCD camera (Photometrics, Tucson, AZ).

### Animal model

Animals were used in accordance with a protocol approved by the University of Arkansas for Medical Sciences (UAMS) Institutional Animal Care and Use Committee. Nude mice (*nu/nu*), 8–10 weeks old, weighing 20–30 g, were procured from a commercial source (Harlan Sprague Dawley, Indianapolis, IN). We also used genetically modified mice expressing human sickle hemoglobin (STOCK Hbatm1Paz Hbbtm1Tow Tg(HBA-HBBs)41Paz/J mice)^41^. The animals were anesthetized by isoflurane and placed on a microscope stage heated to body temperature (38 °C). Most studies were performed on well distinguished, 50–70-μm-diameter blood vessels located 100–200 μm deep in ~300-μm-thick mouse ear. Selected experiments were also performed on mouse mesenteric blood and lymph vessels. The mesentery has an almost ideal structure for verifying cell focusing *in vivo* because it consists of very thin (10–15 μm), transparent connective tissue with a single layer of blood and lymph microvessels assessable by optical technique. The animals underwent minor surgery, but the preparation procedure was safe and did not affect cell properties for at least 5 h. After induction of anesthesia (ketamine/xylazine, 50/10 mg/kg, intramuscularly), animals were laparotomized by a midabdominal incision, and the small-intestinal mesentery was placed on a customized, heated microscope stage and suffused with warmed Ringer’s solution (38 °C, pH 7.4) containing 1% bovine serum albumin to prevent protein loss. To detect PA signals, the ultrasound transducer was acoustically connected to a mouse’s ear or mesentery with warm water.

To optimize acoustic cell focusing *in vitro*, blood samples (1–1.5 mL) were collected from donor mice with the use of sodium citrate as an anticoagulant. Blood samples were fractionated by centrifuging at ~1500*g* for 15 min at room temperature. A thin layer containing WBCs (i.e., buffy coat) was aspirated in a tube. RBC Lysing Buffer (Sigma-Aldrich) was used to remove RBCs as recommended by the supplier. Then WBCs were washed via centrifugation at 1,000*g* for 10 min at room temperature. The pellets were resuspended in phosphate-buffered saline (PBS) to the desired concentration.

### Samples

Gold nanorods (GNRs) that had maximal absorption at 820 nm and were conjugated with antibody against CD45 leukocyte receptors were purchased from Nanopartz Inc. (Loveland, CO). WBCs (leukocytes) were labeled *in vivo* by intravenous injection of ~10^12^ GNRs in 30 μL of PBS into a mouse tail vein.

To enhance PA phenomena in blood and especially lymph flow, 10 μL of indocyanine green (ICG) solution in a concentration of 5 mg/mL in PBS (equal to 2 mg/kg of mouse body weight) was intravenously injected in a mouse tail vein.

Before injection, ICG was filtered through a 0.22-μm-diameter-pore filter. Blood clearance of ICG is biphasic, showing a rapid first phase with a half-life of 10 min and a second phase with a half-life of more than 1 h. The absorption spectrum of ICG lies in the range of 600–850 nm, with maximum absorption in blood at 805 nm. To enhance absorption in blood and lymph flow, we also used silica/gold nanoshells (140 × 15 nm) with maximum absorption near 800 nm, which were provided by the laboratory of nanoscale biosensors at the Institute of Biochemistry and Physiology of Plants and Microorganisms (Saratov, Russia).

White and black (i.e., optically transparent) polystyrene beads with diameters of 6.8 μm and 25 μm were used to verify PA manipulation in PBS and blood *in vitro*.

B16F10 mouse melanoma cells, obtained from the American Type Culture Collection, were cultured at 37 °C in a humidified atmosphere containing 5% CO_2_ with Dulbecco’s modified Eagle medium (DMEM) (Life Technologies/Gibco) supplemented by 10% fetal bovine serum. Melanoma cells were treated with trypsin for 5 min at 37 °C and resuspended in PBS. Cells for injection were concentrated by centrifugation of the initial suspension.

### Ultrasound sources and acoustic measurements

Ultrasound waves were generated by customized piezoelectric transducers (PZT-4) with different widths, thicknesses, and lengths, including transducers measuring 1 × 1 × 8 mm and 1.5 × 2 × 10 mm (Fig. 2a; Supplementary Figs S9 and 10). The transducers were connected to synthesized function waveform generators (model: DG 4062, Rigol Technologies, Oakwood Village, OH; and model: DS340, Stanford Research Systems) through an amplifier (model: F30PV, FLC Electronics AB, Sippedalsvagen, Sweden). The generator produces waves of different frequencies (e.g., 0.2–3 MHz) at a voltage of 7 V. This voltage level was then amplified if necessary up to ten times, producing a final voltage of 70 V.

We also used a Sonicator 730 (Mettler Electronics Corp., Anaheim, CA) with a 1 MHz and 3-MHz ± 10% flat applicator having 5-cm^2^ emitting area (Supplementary Figs 3 and 4). The acoustic radiation, which had a maximal intensity of 2.2 W/cm^2^, was delivered to an animal’s skin through ultrasound gel.

The acoustic resonator (Fig. 1c) consisted of two stainless steel semi tubes with an inner diameters of 0.3 and 0.6 mm and an outer diameter of 0.5 and 1 mm (Supplementary Figs 10 and 11). The transducers were glued to the external surfaces of the semi tubes. Ultrasound gel or water was used for acoustic coupling to biotissue. The imaging of blood vessels was performed through a 0.6-mm-diameter hole in a semi tube. For acoustic coupling, the space between the semi tubes and the mouse ear was filled by water. In one study, we also used a customized resonator consisting of one semi tube and flat substrate and a planar acoustic resonator using a commercial ultrasound source with a flat applicator (above).

The acoustic output was measured with a calibrated ultrasound transducer (e.g., XMS-310, Panametrics-NDT/Olympus, Waltham, MA) and a power meter (UPM DT-1AV, Biomedequip.com). These measurements were performed in a customized water tank with acoustic absorber (rubbers) on the walls to minimize wave reflection and resonance effects. The spatial peak-pulse average acoustic intensity (*I*) was calculated from the equation *I* = *P*^2^/*ρc*, where P is pressure, *ρ* the density of water, and *c* the speed of sound in water. To measure the acoustic parameters in the samples, in particular, inside a capillary, we used a needle-like hydrophone (Precision Acoustics, Dorchester, Dorset, UK) with small-diameter (0.2 mm) PVDF sensor. The temperature rise in a sample was measured with a digital thermometer (H1 98501, Hanna Instruments) placed in the sample immediately after exposure.

### *In vitro* manipulation of beads and cells

*In vitro* studies were performed with a vessel phantom consisting of various-size glass capillaries connected to a syringe pump (model:KDS200, KD Scientific Inc., Holliston, MA) in order to create flow at the required velocity. In the first schematic, we used a glass capillary with a square 100 × 100-μm cross-section and a 360-μm outer dimension (Polymicro Technologies, Inc., Phoenix AZ) located between the glass slide and the cover slip; it was embedded in glycerol to minimize optical distortion and light scattering (Supplementary Figs 1, 2). F. Acoustic waves were produced with rectangular piezoelectric transducers of different sizes (Boston Piezo-Optics, Inc., Bellingham, MA). Transducer and capillary were immersed in water for acoustic coupling. Another capillary measuring 100 mm long has a 0.3-mm inner diameter and a 0.6-mm outer diameter. A thin layer of a 99% glycerol solution (Sigma –Aldrich) was used between the cover glass and capillary. The capillary was filled either with a water solution containing indocyanine green (ICG) in a concentration of 5 mg/mL and 6.8-μm-diameter transparent polystyrene beads or with mouse blood with ICG (10 mg/mL) and 25-μm-diameter black beads. A syringe pump (KDS200) provided flow velocities in the capillary in the range of 0.1–500 mm/s. One end of the capillary was placed in a reservoir containing varying concentrations of nude mouse blood, while the other end was fixed to a syringe applying mild suction.

For PA manipulation, the capillary was irradiated with one or two linear laser beams at wavelengths of 671 or 820 nm (within the absorption spectrum of ICG) (Supplementary Movie 3 and Fig. S13). To explore the possibility for wall-less transportation of the bloodstream across the gap from one capillary to another without blood leakage, we used two round glass capillaries with a 600-μm internal diameter and a 1000-μm outer diameter (Fig. 2c). The two capillaries were separated by an adjustable gap in the range of 0.5–1 mm. A glass capillary with a 1.5-mm inner diameter and 2.25-mm outer diameter was placed over the gap to form of external acoustic resonator (Fig. 2c). The transducer, capillaries, and resonator were immersed in water for acoustic coupling. One capillary was connected to a peristaltic pump (model 13-876-1, Thermo Fisher Scientific, Inc., Pittsburgh, PA) via microtubing. Flow velocity was controlled with a roller clamp. The other capillary tube was connected via microtubing to a 1-mL syringe containing nude mouse blood.

### Cell viability assays

The impact of the ultrasound on live WBCs was estimated with standard cell viability assays (trypan blue, Annexin V-PI, and CellTiter-Glo) according to the manufactures’ procedures. WBCs were extracted from mouse blood and exposed to ultrasound in an Eppendorf container and a glass capillary with a commercial ultrasound source (Sonicator 730) and customized acoustic resonator (Fig. 1c, Fig. S15a–c) at different ultrasound intensities (0.7 W/cm^2^ and 2.2 W/cm^2^) and exposure times (10 s, 5 min, and 30 min). Data are mean ± SD of three or more independent experiments.

### Statistical analysis

A minimum of four animals was used for each experiment. Results are expressed as means ± the standard error of the means. Spearman correlations for which *P* < 0.05 were considered statistically significant. MATLAB, v. 7.0.1 software (MathWorks) was used for statistical analysis.

## Additional Information

**How to cite this article**: Galanzha, E. I. *et al*. *In vivo* acoustic and photoacoustic focusing of circulating cells. *Sci. Rep*. **6**, 21531; doi: 10.1038/srep21531 (2016).

## Supplementary Material

Supplementary Information

Supplementary Movie S1

Supplementary Movie S2

Supplementary Movie S3

Supplementary Movie S4

Supplementary Movie S5

Supplementary Movie S6

Supplementary Movie S7

Supplementary Movie S8

Supplementary Movie S9

Supplementary Movie S10
