# Supplementary material for: In vivo acoustic and photoacoustic focusing of circulating cells
Source: Sci Rep. 2016 Mar 16;6:21531. doi: 10.1038/srep21531 (PMC4793240; doi:10.1038/srep21531)
Supplement: Supplementary Information [file srep21531-s1.pdf]

Supplementary Movie Caption

1. Movie S1. Ultrasound-induced blood focusing *in vitro* (Fig.2b).
2. Movie S2. Acoustic canalization of blood flow in the gap between two capillaries (Fig. 2d).
3. Movie S3. Real-time change of laser beam configuration and location at different wavelengths: 671 nm (red) and 820 nm (blue, coded).
4. Movie S4. PA redirection of 6.8-µm beads through a small slit between the beam and the capillary wall. Laser parameters: wavelength, 671 nm; pulse energy, 1 µJ; pulse rate, 10 kHz. PA contrast agent: ICG.
5. Movie S5. Particle (6.8-µm bead) trapping and oscillation using a linear laser beam at a wavelength of 671 nm, a pulse energy of 5 µJ, and a pulse rate of 10 Hz and ICG as PA contrast agent.
6. Movie S6. Pushing away of a bubble (adhering to a capillary wall) against flow by acoustic waves induced by a linear laser beam.
7. Movie S7. *In vivo* acoustic blood flow focusing in blood microvessel of mouse ear (Fig. 4a).
8. Movie S8. Ultrasound-induced reversible blood stopping in mouse blood vessels (Fig. 4c).
9. Movie S9. *In vivo* PA blood flow focusing in blood microvessels of mouse ear.
10. Movie S10. *In vivo* PA blood stopping in mouse blood vessels (Fig. 5a).

Supplementary Figures

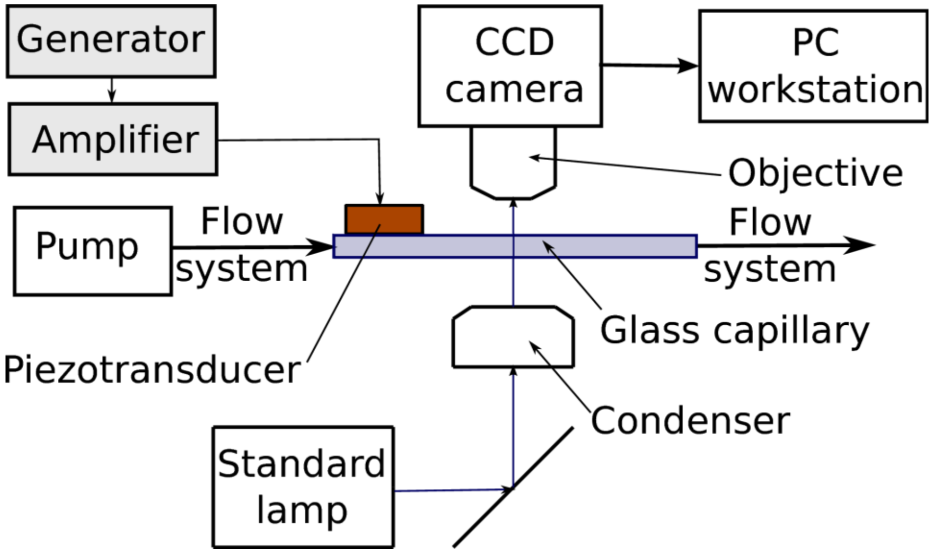

Supplementary Figure 1. Schematic of experimental setup for study of acoustic focusing *in vitro*. The samples are driven through tubes with different diameters (100 μm–3 mm) at a flow velocity in the range of 0.1 mm/s to 50 cm/s.

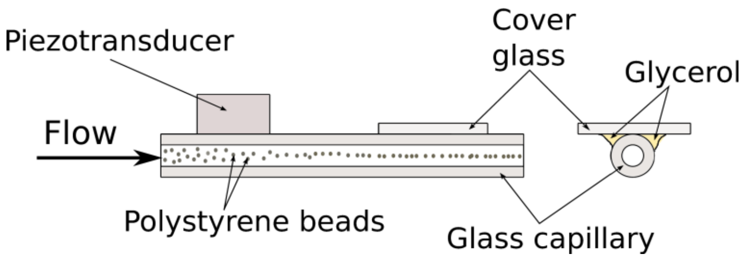

Supplementary Figure 2. Schematic of flow module.

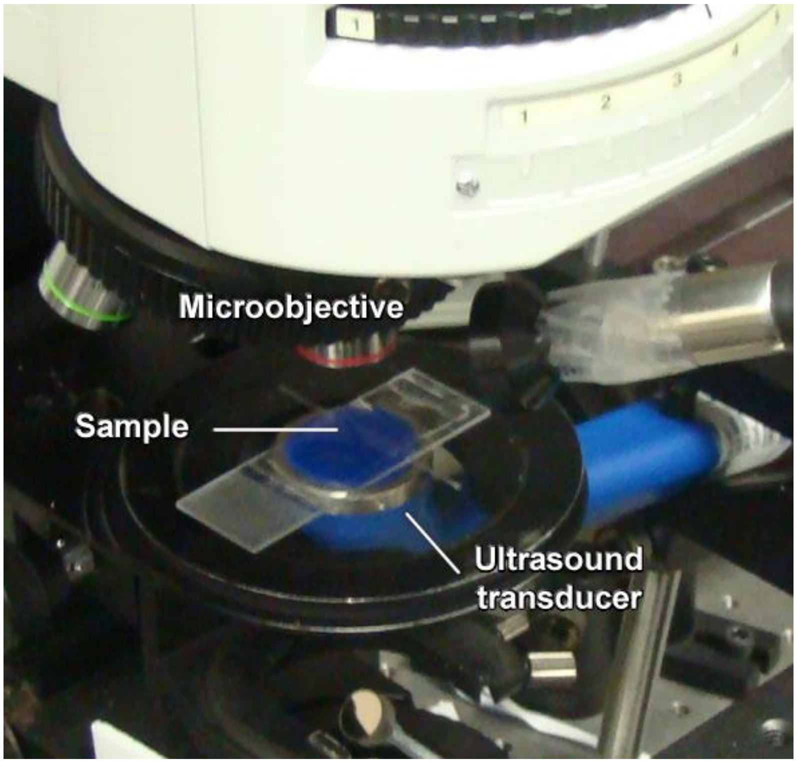

Supplementary Figure 3. Schematic for a study of image-guided ultrasound manipulation of cells and beads in physiological solution and mouse blood *in vitro* using a commercial ultrasound applicator bottom location (Sonicator 730; Mettler Electronics Corp., Anaheim, CA**)**.

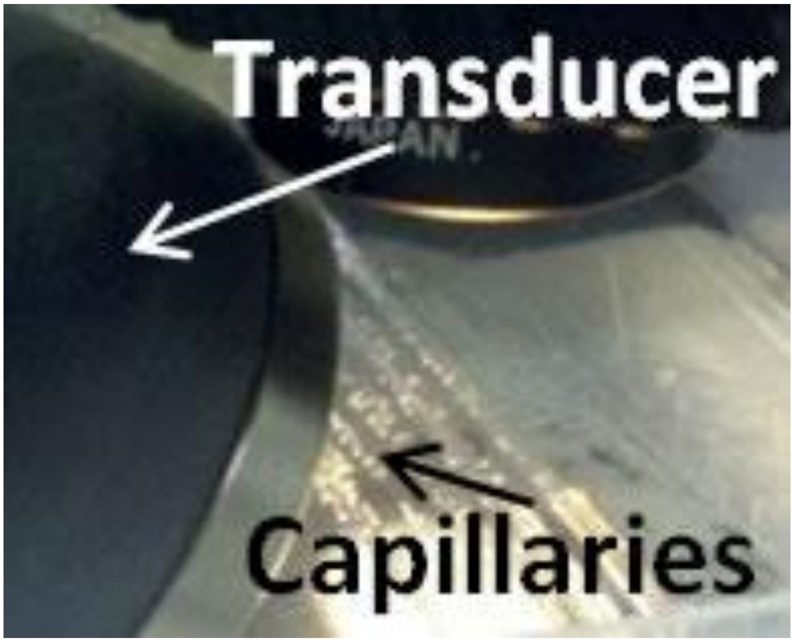

Supplementary Figure 4. Schematic for a study of image-guided ultrasound manipulation of cells and beads in multiple capillaries (parallel acoustic channels) *in vitro* using a commercial ultrasound applicator, upright location (Sonicator 730; Mettler Electronics Corp., Anaheim, CA).

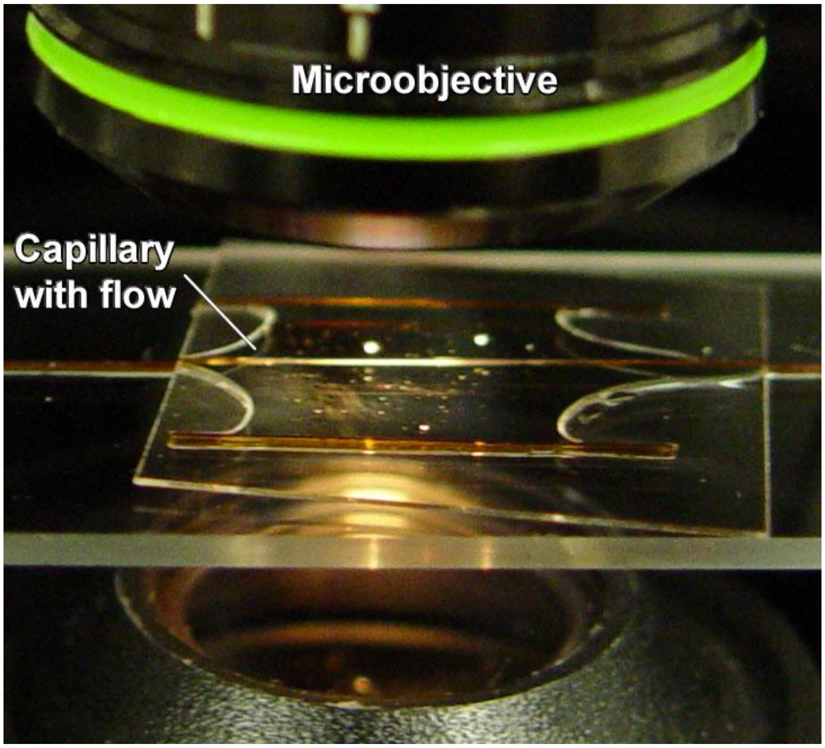

Supplementary Figure 5. *S*chematic for a study of image-guided PA manipulation of cells and beads in physiological solutions and mouse blood flow *in vitro.*

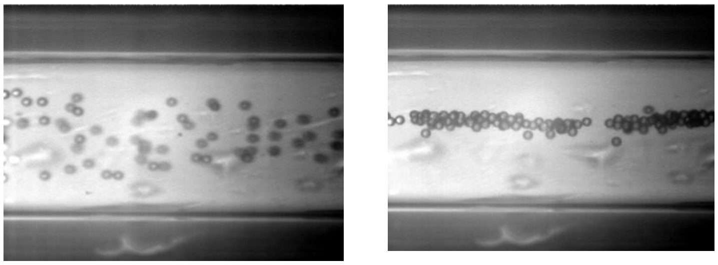

Supplementary Figure 6. Acoustic focusing of 6.8-μm polystyrene beads in 100-μm glass capillary in flow at a velocity of 3 mm/s. Left: before focusing; right: focusing by ultrasound at a frequency of 7.3 MHz and intensity of 1.5 M/cm^2.

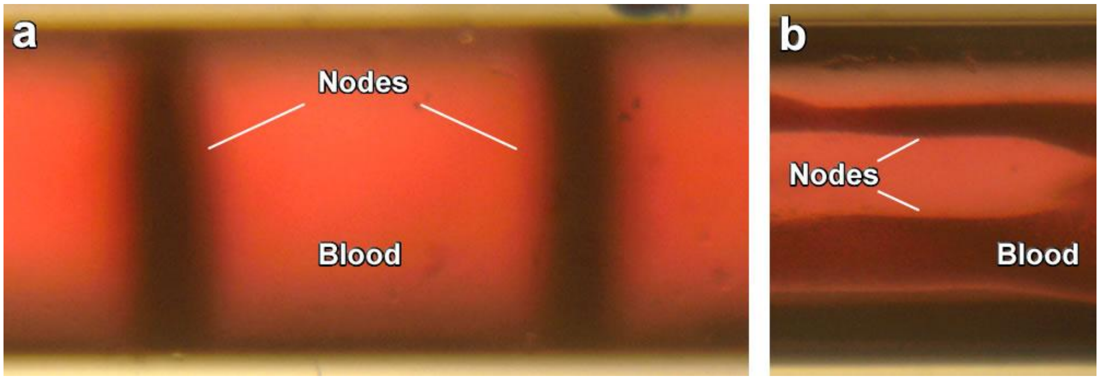

Supplementary Figure 7. ***In vitro*** **blood concentrating in acoustic nodes in 100-µm glass capillary in flow at a velocity of 5 mm/s. (a**) Blood concentration across blood flow. **(b**) Blood concentration along flow in two nodes. The fundamental ultrasound frequency, 7.28 MHz; intensity, 0.7 M/cm^2. The ultrasound waves were formed with customized piezotransducers.

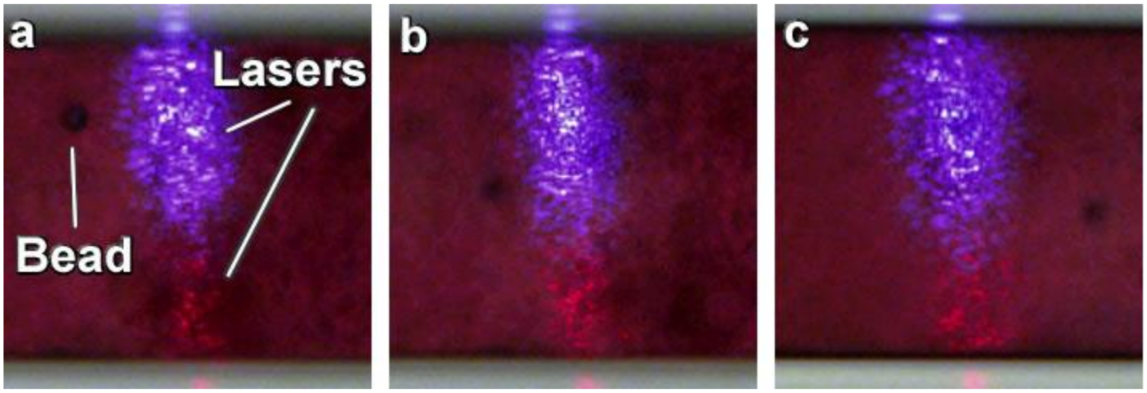

Supplementary Figure 8. (a-b) ***In vitro*** **PA focusing of 25-µm polystyrene beads in single file in blood flow between two linear laser beams.** Laser parameters: wavelength, 671 nm (bottom) and 820 nm (top); beam sizes at the entrance of the capillary, 4.5 µm x 80 µm (671 nm) and 8 µm x 90 µm (820 nm); energy fluence, 50 J/cm^2 (671 nm) and 80 J/cm^2 (829 nm). The laser beam images are shown after passing of capillary and blood flow leading to image blurring due to light scattering. Flow module consists of a circular glass capillary with an inner diameter of 300 µm and blood flow velocity of 3 mm/s.

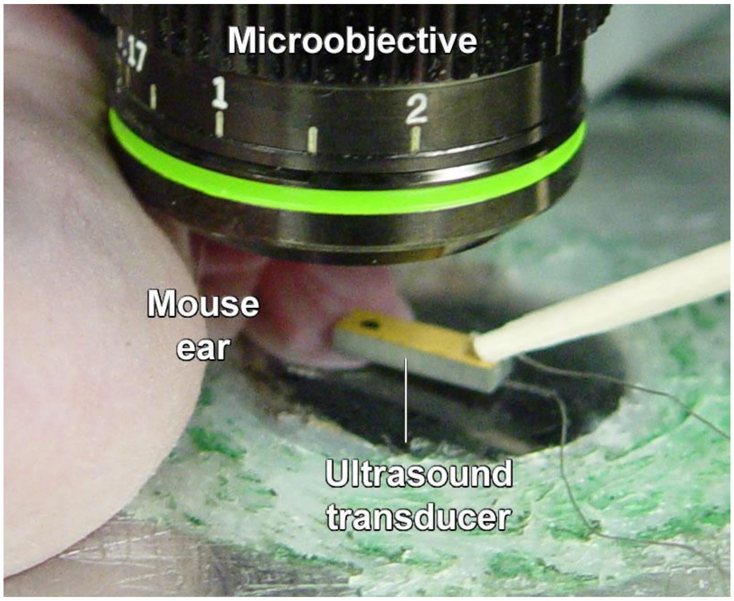

Supplementary Figure 9. ***In vivo*** **schematic of acoustic focusing of blood flow in mouse ear microvessels.**
The standing ultrasound waves were formed in the flat resonator with a customized piezotransducer.

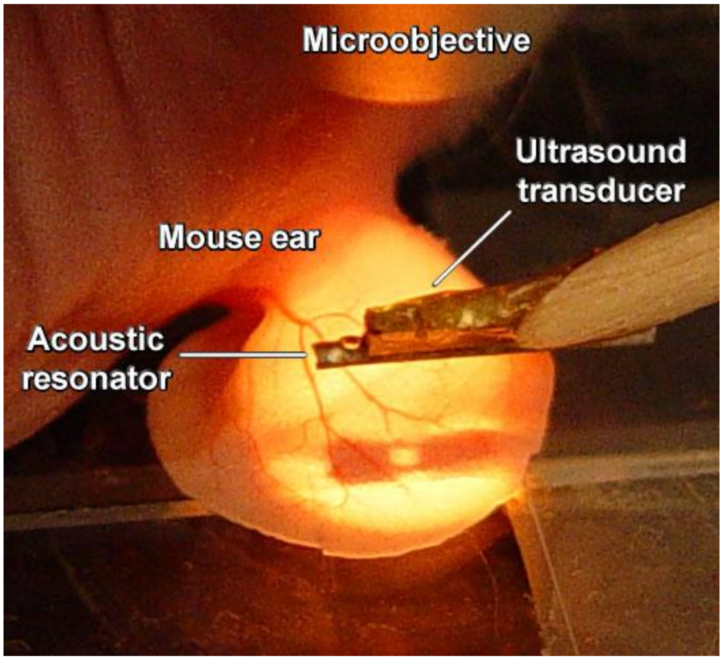

Supplementary Figure 10. ***In vivo*** **schematic of acoustic focusing of blood flow in mouse ear microvessels using ultrasound waves in an external acoustic resonator.** The resonator consisted of two stainless steel semitubes or one semitube and a flat substrate. One semitube had a hole for optical imaging.

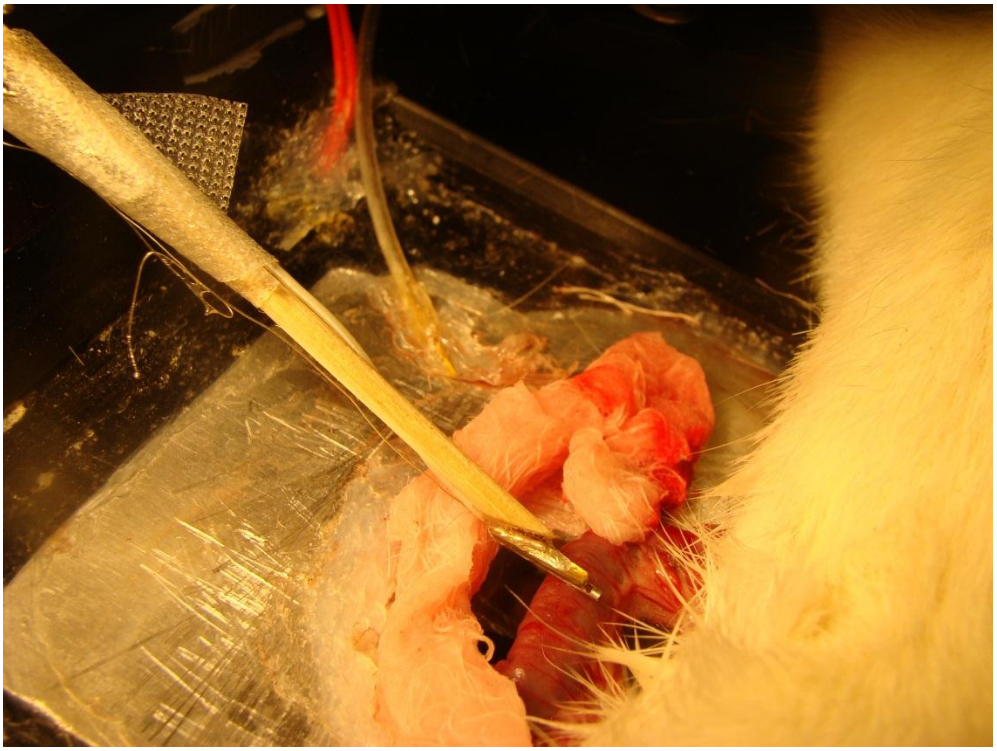

Supplementary Figure 11. ***In vivo*** **schematic of acoustic focusing of blood and lymph flow in mouse mesentery vessels using ultrasound waves in the external acoustic resonator.** The resonator consisted of one flat acoustic “mirror” and one semitube with a hole for imaging.

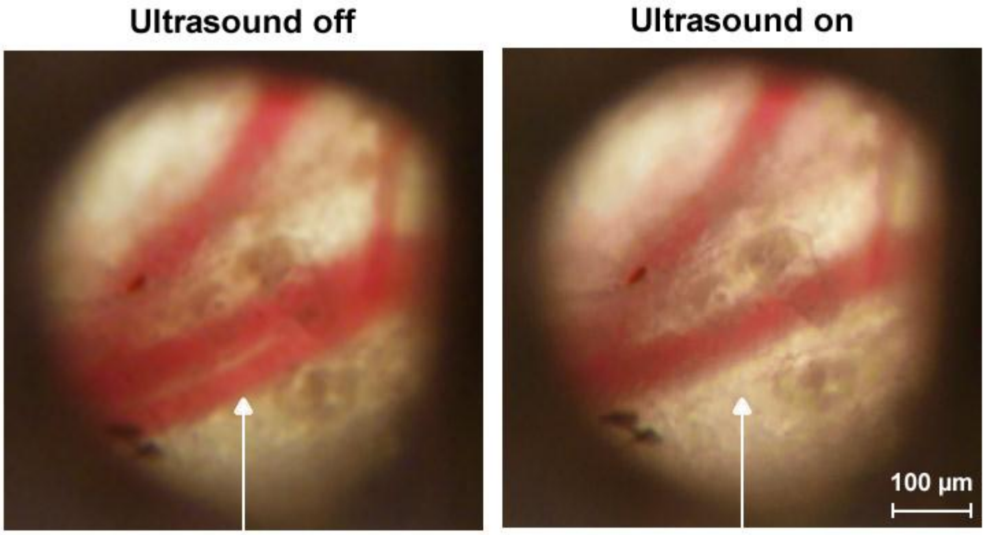

Supplementary Figure 12. *In vivo* acoustic focusing of blood flow in mouse ear microvessels using the acoustic resonator consisting of two semitubes (Fig. 1a and Fig. S10). Left: normal flow (control, no ultrasound). Right: acoustic focusing of blood flow selectively (bottom vessel) into a tiny stream (single file) with a diameter of 5-10 µm. Ultrasound parameters: frequency, 1.3 MHz; intensity, 0.6 W/cm^2, flow velocity, ~4 mm/sec.

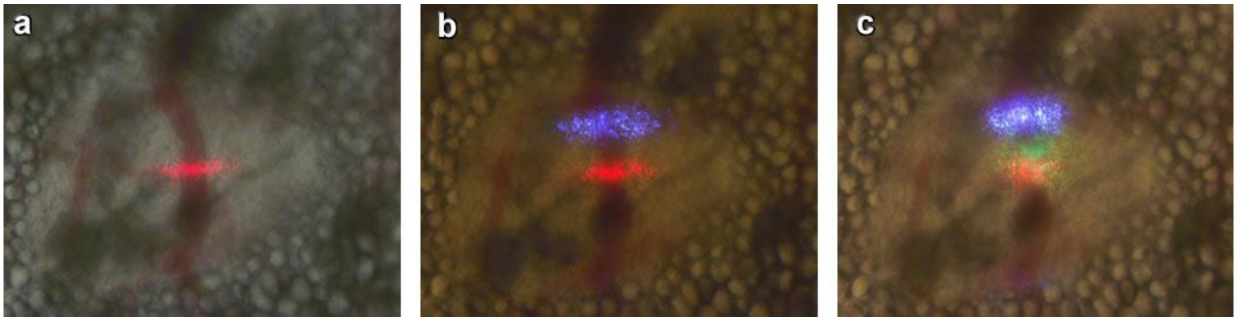

Supplementary Figure 13. Images of one (a), two (b) ,and three (b) linear laser beams at wavelengths 532 nm (green), 671 nm (red), and 820 nm (blue, coded) after passing 300-µm-thick mouse ear tissue with 40-µm blood vessels. The beam size at the entrance of superficial blood vessel is ~4.5 x 80 µm at 671 nm.

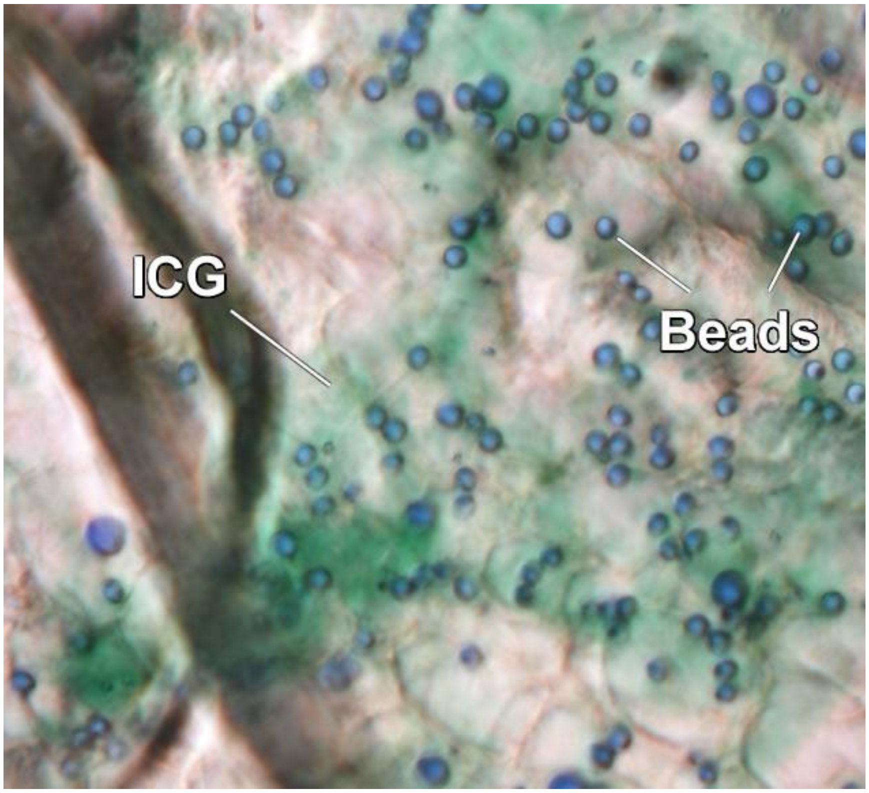

Supplementary Figure 14. Image of mouse mesentery 1 h after intravenous injection of 6.8-µm polystyrene beads and indocyanine green into a mouse tail.

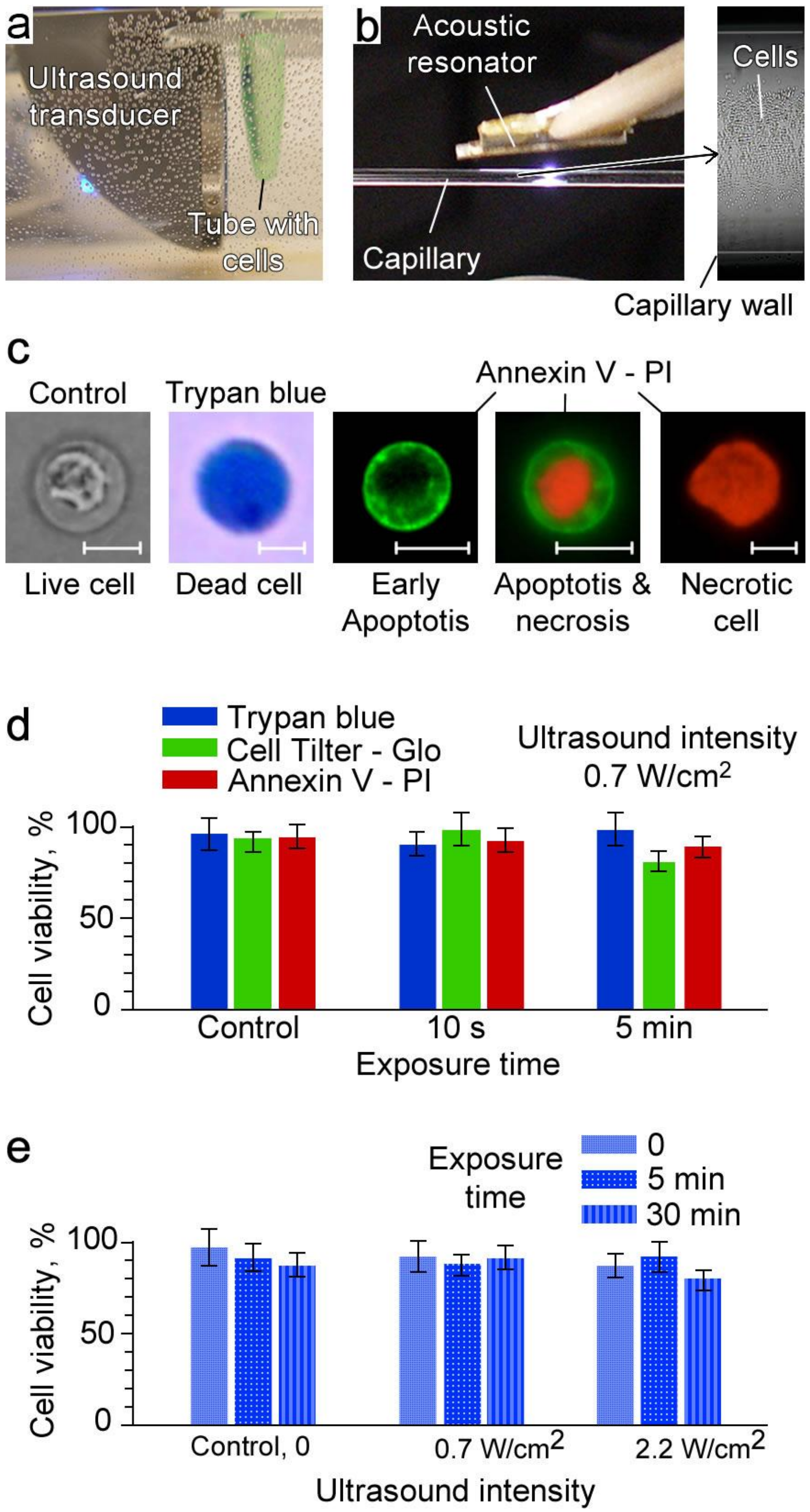

a
Ultrasound transducer
Tube with cells
b
Acoustic resonator
Capillary
Cells
Capillary wall
c
Control
Trypan blue
Annexin V - PI
Live cell
Dead cell
Early Apoptotis
Apoptotis & necrosis
Necrotic cell
d
Trypan blue
Cell Tilter - Glo
Annexin V - PI
Ultrasound intensity 0.7 W/cm2
Cell viability, %
100
50
0
Control
10 s
5 min
Exposure time
e
Exposure time
0
5 min
30 min
Cell viability, %
100
50
0
Control, 0
0.7 W/cm2
2.2 W/cm2
Ultrasound intensity

Supplementary Figure 15. Effect of ultrasound on the viability of white blood cells (WBCs). (a) Schematic of cell viability test in an Eppendorf tube using a commercial ultrasound source (Sonicator 730) with a 2-cm-diameter applicator (Figs. S3, 4). The transducer and tube were housed in a water tank. (b) Schematic of cell viability test in a 300-μm-diameter glass capillary with a wall thickness of 150 μm using a customized acoustic resonator with a PZT transducer (Figs. 1c, S12). Cells were infused in a capillary and collected after exposure in an Eppendorf tube. (c) From left to right: examples of optical images of intact, dead (filled with with trypan blue), apoptotic, apoptotic-necrotic, and necrotic cells tested with Annexin V-PI assay. (d) Viability of WBCs after ultrasound exposure in an Eppendorf tube at an intensity of 0.7 W/cm^2 and exposure times of 10 s and 5 min measured with three viability assays: trypan blue, Annexin V-PI, and CellTilter-Glo. (e) Viability of WBCs after ultrasound exposure in a capillary tube with an inner diameter of 300 μm using a customized acoustic resonator (see Fig. S10) at an ultrasound intensities of 0.7 W/cm^2 and 2.2 W/cm^2. Data are means ±SD of three or more independent experiments.

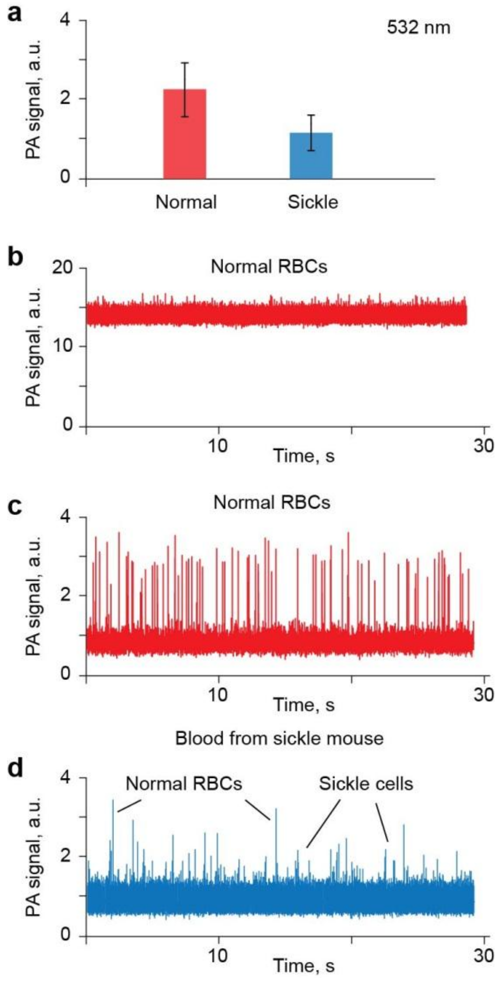

Supplementary Figure 16. PA detection of normal RBCs and sickle cells from control mice and genetically modified mouse model expressing human sickle hemoglobin. (**a**) Average PA signal amplitudes from normal RBCs and sickle cells. Laser parameters: wavelength, 532 nm; fluence, 60 mJ/cm^2 , laser beam diameter, 10 µm. These data were obtained by irradiation of each cell in a slide with a circular laser beam covering the whole cell. They show that on average PA signal amplitudes from individual normal RBCs are ~2 times larger than those from sickle cells. (**b**) Control PA signal trace from undiluted normal blood in flow in a 50-µm-diameter capillary tube at a velocity of 5 mm/s. The ultrasound transducer (model XMS-310 with a 10-MHz frequency band; Panametrics) was attached to a capillary tube through thin layer of ultrasound gel. This trace is relatively stable without transient PA peaks because of the overlapping of PA signals from many RBCs in the detection volume. (**c**) PA signals from individual RBCs in normal blood in flow in a capillary tube as in (**b**). However, to exclude temporal overlapping of PA signals from many RBCs as in (**b**), the blood sample was diluted 1000-fold, thus providing single-file flow through the 10-µm-wide linear laser beam. Some

variations in PA signal amplitudes are associated with natural RBC heterogeneities in size and the change of the positions of cells in the cross-section of tube. (**d**) PA signal trace from blood from a mouse model of human sickle disease. The flow parameters are the same as in (**c**). Laser parameters were (**c,d**): wavelength, 532 nm; fluence, 40 mJ/cm^2; pulse rate, 10 kHz; and pulse width, 10 ns. Data in (**c**) demonstrate the feasibility of PA detection of individual RBCs in single-file flow. Comparison of PA data in (**c**) and (**d**) indicates the possibility of identifying cells producing PA signals with different amplitudes. In particular, the PA signal trace in (**d**) shows a higher number of signals with lower amplitude, presumably from sickle cells, compared to the trace in (**b**) obtained from normal cells.

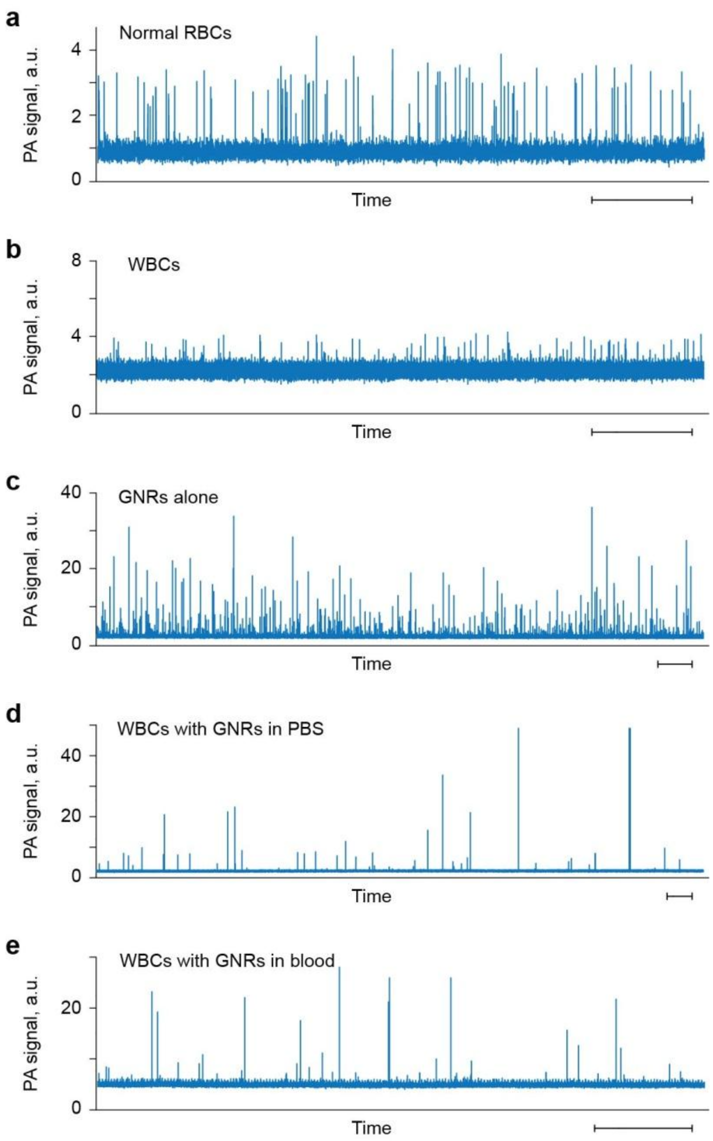

Supplementary Figure 17. PA signal traces from normal RBCs, WBCs, gold nanorods (GNRs) , WBCs with GNRs, and mouse blood with targeted WBCs. (**a**) PA signal trace from normal blood in flow in a 50-µm capillary tube at a velocity of ~3 mm/s (similar to the trace in Supplementary Fig. 16a). To exclude temporal overlapping of PA signals from many RBCs in the detection volume, the blood sample was diluted 1000-fold, thus providing single-file flow through a 10-µm-wide linear laser beam. The ultrasound transducer (model XMS-310 with a 10-MHz frequency band; Panametrics) was attached to a capillary tube through thin layer of ultrasound gel. Some variations in PA signal amplitudes are associated with natural RBC heterogeneities in size and changes of positions of cells in the cross-section of the tube. (**b**) PA signal trace from WBCs in flow with parameters as in (**a**). WBCs were extracted from mouse blood according to a well-established protocol (Methods) and introduced into flow at a concentration similar to that of RBCs in (**a**). Comparison of traces in (**a**) and (**b**) shows that WBCs produce much smaller-amplitude PA signals than RBCs because of the stronger absorption by hemoglobin in RBCs than by cytochromes in WBCs as natural PA contrast agents. (**c**) PA signal trace from GNRs with maximum absorption at 670 nm alone in PBS flow at a

concentration of 10^9/mL. This trace shows that many GNRs randomly distributed in PBS produce many fluctuating PA signals due to the different numbers of GNRs in the detection volume at each time and to the possible influence of small aggregates. (**d**) PA signal trace from WBCs labeled with GNRs in PBS flow. (**e**) PA signal trace from WBCs labeled with GNRs in mouse blood flow. Labeled WBCs were spiked with mouse blood at a concentration similar to (**d**) condition. Comparison of traces in (**d,e**) shows that labeled WBCs produce stronger signals than GNRs in PBS and signal-to-noise in blood are lower because of background absorption by blood. Laser parameter (**a-e**) were: wavelength, 671 nm; fluence, 60 mJ/cm^2; pulse rate, 10 kHz; and pulse width, 10 ns. Time scale, 5 sec.
